# Development of a school program for vaping and smoking prevention and protocol for a cluster randomized controlled trial in fifth grade students

**DOI:** 10.1038/s41598-026-45720-w

**Published:** 2026-05-07

**Authors:** Marina Hinssen, Julia Kohn, Josef Mohammad, Valentin Jakob Vecera, Gertraud Stadler

**Affiliations:** CharitéCenter for Prevention, Health and Human Sciences, Charité ─ Universitätsmedizin Berlin, Freie Universität Berlin, Humboldt-Universität Zu Berlin, and Berlin Institute of Health, Augustenburger Platz 1, 13353 Berlin, Germany

**Keywords:** Health care, Medical research, Psychology, Psychology

## Abstract

This article presents the intervention development study and protocol for a longitudinal cluster-randomized controlled trial of a school-based vaping and smoking prevention program based in Germany. E-cigarette use is rapidly increasing among adolescents who have never smoked, yet well-evaluated prevention interventions are lacking. Although school-based prevention programs are moderately effective, they are often difficult to implement in schools facing high structural burden. Using Intervention Mapping, we developed a vaping and smoking prevention program intended for a broad range of schools, including lower-SES settings. Development drew on qualitative interviews with students (*N* = 62) and experts (*N* = 54), followed by feasibility testing in 13 classrooms with pre- and post-intervention surveys *(N* = 92). The resulting intervention comprises three classroom sessions delivered by trained facilitators. Interview and feasibility data supported: (i) lowering the prevention target age to Grade 5; (ii) emphasizing e-cigarettes, resistance skills, social norms, and stress management; (iii) adapting materials to improve fit with schools serving lower-SES student populations; and (iv) the intervention’s high acceptability and feasibility, suggesting readiness for full-scale evaluation. The intervention will next be tested in a cluster-randomized controlled trial with at least 1500 students across 26 Berlin schools, randomized (1:1) to intervention or active control group.

**Supplementary Information:**

The online version contains supplementary material available at 10.1038/s41598-026-45720-w.

## Introduction

Smoking is one of the leading behavioral risk factors for disease and premature mortality worldwide^1^. Tobacco consumption harms nearly every organ of the body^2^, and smokers experience lower health-related quality of life, including poorer physical functioning, vitality, and mental health^3^. Concurrently, the use of electronic cigarettes has increased since their introduction to numerous national markets around 2006, particularly among adolescents and young adults^4–7^. In many countries, e-cigarettes have become more prevalent among schoolchildren and adolescents than traditional tobacco cigarettes^4,6^. This pattern is also observed in Germany: among 9–17-year-olds, lifetime cigarette use was reported by 6% of girls and 7% of boys, whereas lifetime e-cigarette use was reported by 15% and 19%, respectively^8^. The rising number of young individuals who never smoked cigarettes and who are now vaping regularly is a matter of concern, as it suggests that these novel products are attracting young people who would not otherwise smoke^7^. Despite being promoted as a less harmful alternative to conventional cigarettes^9^, constituents of e-liquids and inhaled aerosols have considerable addictive potential^10^, are carcinogenic, harm lung tissue and airways^11^ and place acute strain on the cardiovascular system^12^.

In addition to the surge in vaping, differential consumption trends among population subgroups, defined by characteristics such as gender and family income, merit attention. Firstly, a persistent–or even widening–gap in nicotine use exists among individuals from lower and higher socioeconomic status (SES) backgrounds across the lifespan^13^: Being born into a lower SES family^14,15^, growing up in a deprived region^16^, or attending a lower-SES school^15^ increases the likelihood of being surrounded by smoking family and friends, initiating smoking and vaping, and becoming addicted to nicotine^14,15^. Recent studies from Germany^16^ and the U.K ^17^, also suggest that vaping is more concentrated among lower SES populations. In a recent German survey of adolescents, those growing up in the most deprived regions had between 1.6- and 2.2-fold higher odds of cigarette use, e-cigarette use, and poly use than those in the most affluent regions^16^. Second, while males continue to exhibit higher smoking prevalence and smoking-attributable mortality in the overall population, evidence suggests that these disparities are narrowing and have closed or even reversed among school-aged children in high income nations^1,4,8^.

### Interventions for vaping and smoking prevention

It is widely acknowledged that schools are a favorable setting for prevention efforts, given that the majority of children and adolescents from various social strata spend more time at school than in any other setting outside their homes^18^. A Cochrane review of 134 studies supports this assertion, demonstrating that school-based smoking prevention programs have been shown to reduce the risk of students initiating smoking by an average of 12% over the longest follow-up period (OR 0.88)^19^. Effective programs were characterized by training youth in general personal and social skills (e.g., problem solving, critical thinking, coping with stressors and unpleasant emotions), ideally combined with increasing their awareness of negative social influences and skills to resist them (“combined social skills and social influence curricula”, OR 0.50). Regarding e-cigarette use, there is a dearth of high-quality research (RCTs) on the long-term preventive effects on behavioral outcomes, which precludes the drawing of firm conclusions. However, the initial findings also point to the promising effects of incorporating social and emotional competence or influence elements in programs^20,21^. Theoretically, these competencies are regarded as *life skills* that are vital not only for the avoidance of substance use, but more broadly for adaptive coping with the demands of everyday life and developmental tasks during adolescence^22,23^. Therefore, it is hypothesized that life skills function as protective factors, thereby reducing the probability of adolescents engaging in substance use when confronted with adversity^23,24^. Substance use prevention programs grounded in the life-skills-approach are generally implemented by teachers and professional educators who have undergone specialized training^25^. They are typically administered over the course of 12 to 15 lessons per school year, with the program’s duration extending for two or more consecutive school years^25^.

However, challenges to implementing health promotion programs in schools have intensified worldwide due to teacher shortages, COVID-19-related learning losses and competing core-curricular demands^26,27^. There is an evident need to refine and reduce complex interventions to their essential components in order to create an implementable and cost-effective remedy to the trend of declining children’s physical and mental health and wellbeing^28^. A viable starting point would be to examine effective school programs in respect to the key mechanisms through which they promote positive health behaviors. This approach may facilitate the identification of particularly influential, modifiable behavioral determinants and strategies that are critical to effective smoking prevention^29^. However, data on mechanisms mediating effects are scarce across smoking prevention and health behavior change interventions as a whole^29^.

Despite the evidence supporting the efficacy of school-based smoking prevention programs, modest effect sizes indicate the potential for improvement in our intervention strategies^19^. One contributing factor may be the failure to consider heterogeneity among students. Individual differences in risk and protective factors may shape their intervention needs and, consequently, moderate the effectiveness of prevention programs. This may be particularly true for social determinants of health (SDOH), such as student gender, family income, religion/wordview, ethnicity/race, and health status. These factors are associated with varying degrees of exposure to health compromising conditions, including material deprivation, psychosocial strain, and negative health behavior (i.e., smoking)^30,31^. For example, girls tend to imagine that smoking could help them manage negative emotions, and low self-esteem and stress are important risk factors for their smoking initiation^32,33^. In contrast, boys tend to have positive expectations about smoking, such as experiencing stimulation, a “buzz,” or a favorable social outcome, e.g., appearing more masculine^32,33^. Low SES youth and members of minority groups may also experience elevated levels of stress and a range of unique stressors, including stressful life events, financial stress, and negative family relationships^34–36^. These factors, in turn, are associated with increased risk of smoking and developing nicotine dependence^34–36^.

Although individual student needs may vary, existing school-based programs and their evaluation studies often employ a “one-size-fits-all” approach. They typically target entire classrooms at once and evaluate effectiveness by assessing the average smoking behavior of all participating students.

### Study aims for the intervention development study and the trial protocol

The aim of this article is twofold: First, we report the participatory development of a school-based prevention program addressing vaping and smoking, tailored to the context of schools serving students from lower socioeconomic backgrounds. Second, we present the protocol for a longitudinal cluster-randomized controlled trial designed to evaluate the program’s effectiveness in strengthening students’ intention to remain nicotine-free and in reducing nicotine initiation and consumption. In addition, the study will examine the mediating mechanisms of program effects to identify which intervention components are critical to the success of vaping and smoking prevention. We will also explore heterogeneity in risk and protective factors, intervention effects, and mechanisms of action according to students’ social determinants of health. The results may therefore provide insights into group-specific needs and inform differentially effective prevention strategies.

## Methods

### Ethics approval and consent to participate

We have received approval for this research by the ethics committee of the Charité – Universitätsmedizin Berlin (Reference Number Development Study: EA2/222/22; Reference Number Evaluation Study: EA2/225/23) and the Berlin Senate Department for Education, Youth, and Family. Any substantial changes to the protocol will be communicated to the respective authorities in the form of formal amendments and documented via updates of the pre-registration for this project^37^. All methods were and will be performed in accordance with relevant guidelines and regulations. Written informed consent was obtained from all participants; for student participants, informed consent was obtained from both the students and their legal guardians.

### Development study

#### Methods and frameworks

**Fig. 1:**
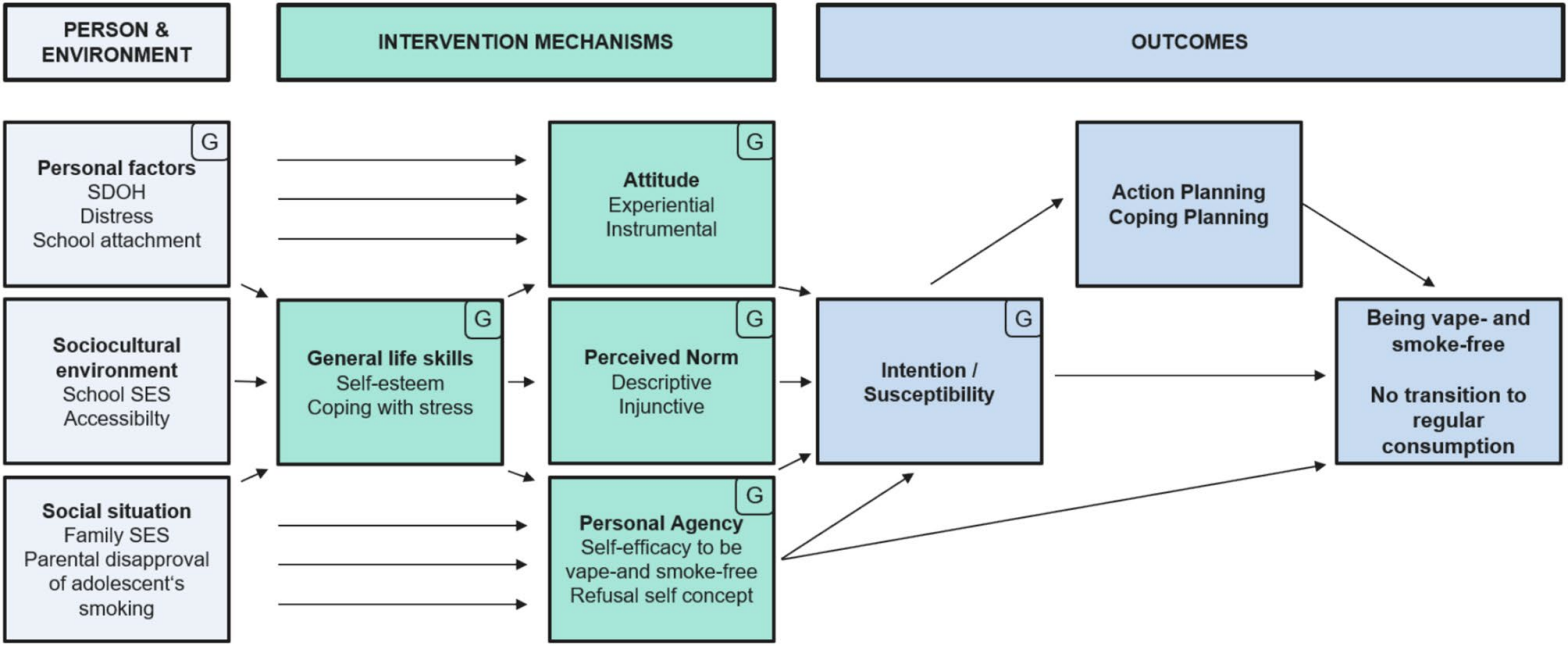
Logic model for the vaping and smoking prevention intervention. Behavioral determinants marked with the letter “G” indicate hypothesized gender differences in the absolute levels and/or predictive value of those determinants for the outcomes (see pre-registration file for more details and hypotheses). SDOH, socal determinants of health; SES, socioeconomic status.

We developed the intervention using a stepwise, iterative, and participatory approach. To complete development, feasibility testing, and trial preparation within a targeted timeframe of 12 months, we pragmatically followed the Intervention Mapping Adapt approach for adapting evidence-based interventions to new settings and populations^38^. To identify highly relevant and modifiable determinants of vaping and smoking to address in the intervention (i.e., to conduct the needs assessment) and to develop our program theory (i.e., our assumptions about the pathways of program effects; logic model of change, Fig. 1)^39^, we drew on the Integrated Behavioral Model ^40^ and other influential health behavior, substance use, and child development theories ^24,41–45^, as well as existing evidence on the determinants (correlates/predictors) of smoking behavior [e.g.,^46^], school-based smoking prevention programs that have been shown to be effective in high-quality evaluations, and studies examining the mechanisms that mediate the effects of these programs. We searched the relevant literature from 06/2022 to 10/2022 using PubMed and Central search engines, as well as the Xchange Prevention, Green List Prevention, and RTIPs (U.S. National Cancer Institute’s Research-Tested Intervention Programs) registries. Reviews and meta-analyses were included if published after 2000; trials were included if listed in the most recent Cochrane review of smoking prevention trials published from 1966 to 2012^19^ or published after 2012 in the above databases. In addition, after specifying the determinants of vaping and smoking to be addressed in the program (i.e., change objectives)^39^, we consulted the Behavior Change Wheel (BCW)^47^ and the Theory and Techniques Tool (TaTT)^48^ to select the intervention techniques (i.e., Behavior Change Techniques as defined in the BCT Taxonomy v1 (BCTTv1,^49^) and to describe the content of our intervention.

#### Data collection

Qualitative and quantitative data collected in spring and summer 2023 informed the iterative process across three areas: a) needs assessment and subsequent logic model refinement, b) development of intervention concept and materials and c) feasibility testing. We briefly give an overview of aims, content and context of data collection. Further details on the interview procedures, including the interview prompts, are presented in the Supplementary 1; Notes S1 and S2.

Based on common practice in most existing smoking prevention trials [^19^], the intervention was initially considered to target students aged 12–13. This corresponds to Grade 7 students in Germany, and, internationally, an age group with high rates of first experimentation with e-cigarettes and cigarettes^4^. However, early stakeholder consultations suggested that initiation of vaping and smoking often occur earlier, particularly in schools with lower socioeconomic status. Therefore, the intervention was developed with a target group of grade 6 children (11–12 years old in Germany).

All participating 6th grade students (*N* = 62, mean age *M* = 11.65, *SD* = 0.68, 51.61% female) and their teachers (*n* = 4) were recruited at two lower socioeconomic status (SES) schools classified as having high and very high sociostructural burden according to the Berlin school classification index (SenBJF Education Statistics Division;^50^). Additional stakeholders and other teachers were recruited through project collaboration partners, professional networks, and personal contacts. Resultingy, stakeholders participating in semi-structured interviews (*N* = 7) comprised four teachers (three from the recruitment schools, one from a low-burden school), one social worker working with underserved youth, and one teacher training manager focused on educational equity. Stakeholder field exchanges *(N* = 47) involved governmental (*N* = 15) and school-level prevention coordinators (*N* = 20), youth health interventionists (*N* = 8), and researchers (*N* = 4) specializing in education, school psychology, addiction prevention, or didicatics.

#### Needs assessment and logic model refinement

*Needs assessment: Student (STU) interviews*. Semi-structured needs assessment interviews were conducted with a subgroup of the student sample (*n* = 33 of *N* = 62) during school hours in a separate room. Interviews were audio-recorded and documented using structured templates. The aim was to identify key determinants of vaping and smoking to inform the intervention logic model. Interviews explored students’ knowledge and experiences with nicotine products. A *Salient Belief Elicitation Approach* based on the Integrated Behavioral Model and the Reasoned Action Approach^40,51^ was used to assess affective, normative, and control beliefs related to being smoke-free. Additional topics included peer influence, access to nicotine products, gender differences, school policies, exposure to advertising and social media.

*Needs assesment: Stakeholder (STA) interviews and exchanges.* Semi-structured needs assessment interviews with school stakeholders (*N* = 7; *n* = 2 on-site *n* = 5 online) were conducted outside school hours and documented through interviewer notes. These interviews addressed schools’ organizational and teachers’ capacities to implement or support prevention efforts. In addition, they mirrored the student interviews by examining the stakeholders‘ perspective on student vaping and smoking behavior and its determinants, including gender differences, and group-specific needs.

Additional insights were gathered through stakeholder field exchanges *(N* = 47; duration 60–180 min). These included group discussions during two prevention-related events with governmental and school-level prevention coordinators (one online event with *n* = 20 participants, one in-person event with *n* = 15 participants), online meetings with researchers and health prevention interventionists (*n* = 7; 60 min duration) and discussions with health interventionists following observation visits in school (*n* = 5; 20–60 min duration). These exchanges expanded on the stakeholder interviews by addressing the same topics while focussing on participants‘ specific areas of expertise. For example, discussions with researchers and interventionists focused on the feasibility of study procedures in the target setting, such as obtaining parental and student consent and pseudonymization approaches used to match students‘ longitudinal data. Field exchanges were documented using field notes.

#### Material and concept review

*Review of materials and concept: Student (STU) interviews.* Material and concept review interviews gathered feedback from a second subgroup of the student sample (*n* = 29 of *N* = 62) on workshop content and materials. Interviews were conducted either in a simulated intervention format (*n* = 15) in which the interviewer delivered a condensed version of the workshop using draft slides and materials or as a *post-lesson review* (*n* = 14) following feasibility test sessions. Sessions combined content presentation with think-aloud prompts, and interview foci were adapted iteratively to emerging development needs.

*Review of materials and concept: Stakeholder (STA) interviews and exchanges.* Stakeholders (*N* = 7 interviews; *N* = 47 exchanges) continued to give input on the intervention concept and draft materials after the needs assessment described above. This included reflections following presentations of the intervention concept or direct experience with the draft program during feasibility testing. Discussions began with spontaneous impressions and were followed by targeted questions on feasibility, acceptability, and program design. An exception were exchanges with practitioners (*n* = 5) following on-site observations of their school-based prevention activities. These discussions focused on reflections regarding the observed intervention and its implications for the design of the tobacco and nicotine prevention program.

#### Feasibility testing (FEA): Pre- and post-intervention online questionnaires

Further into the development process, the materials were structured into three program blocks, each designed to last approximately 90 minutes. *Core Workshop 1* included an expert quiz, information on immediate and long-term consequences of vaping and smoking (e.g., health, fitness, mental well-being, effects on close others), and a smoking experiment. The workshop was pretested in eight classes, with 92 students providing informed consent for survey participation. *Core Workshop 2* focused on refusal strategies with role-play practice, normative feedback on vape- and smoke-free peers, critical discussion of marketing and social media influences, and creating a vape- and smoke-free advertisement. The workshop was pretested in three classes, with 42 students providing informed consent and survey responses. An additional *Life Skills Training* addressed personal resource recognition, problem-solving, and coping with unpleasant emotions, and included a breathing exercise. The workshop was pretested in two classes, with 59 students participating in the surveys.

Students completed pre–post questionnaires assessing vaping and smoking behavior, targeted determinants from the logic model, and intervention acceptability. Teachers (*N* = 4) completed post-lesson surveys assessing program acceptability and feasibility. The primary outcome was student and teacher-reported satisfaction^52^ with the program. This was assessed using items such as: “All in all, I really liked the program“ (1 = *strongly disagree*, 5 = *fully agree*; students and teachers), “Would you accept the implementation of the program in your class again?“ (yes/ no, with optional explanation; teachers), and “Should the program also be offered to other students at your school?” (yes/no; students). Teachers additionally reported on the feasibility of implementation, including the perceived ease of integrating the program into regular teaching and routine school practice, rated on a 5-point Likert scale (*1* = *strongly disagree*, *5* = *strongly agree*).

#### Data analysis

*Interview and exchange data.* Qualitative analyses served two distinct aims –addressing research gaps and intervention development–  and therefore differed in depth and timing. The first aim was to systematically generate insights into needs for tobacco and nicotine prevention in lower SES schools, with particular attention to gender differences and new nicotine products. For this purpose, audio-recorded needs assessment interviews with students were transcribed verbatim and independently analysed by two researchers not involved in intervention development, using reflexive thematic analysis^53^. Results will be published separately, including detailed theme descriptions and illustrative quotations.

The second aim was concrete guidance for intervention development. Given the one-year development timeframe, a pragmatic analytic approach was applied. Immediately after each needs assessment and material/concept review interview,  a structured documentation template was completed. The needs assessment template captured interviewees’ reported beliefs as well as perceived barriers and enablers to remaining nicotine-free. The material and concept review documentation template captured structured feedback on each intervention component.

The intervention development lead reviewed needs assessment templates to identify frequently reported beliefs and differences between students from high versus very high sociostructural burden schools, and between female and male students. Based on observed patterns, prevention-relevant needs were inferred, prioritised, and systematically translated into the logic model and intervention components.

Material review interviews were conducted in iterative blocks with adapted content and procedures (e.g., students participated in intervention simulations or in post-lesson interviews, stakeholders received presentations of intervention concepts or were interviewed after experiencing the feasibility testing in school). After each interview block, documentation was systematically reviewed to derive adaptation needs for specific intervention components, informing successive refinements of the logic model and workshop materials.

*Survey data.* Student data were screened for completion time and missing responses and analysed descriptively (means, frequencies). Internal consistency (Cronbach’s α) was assessed for multi-item measures, composite scales were constructed, and construct validity was examined via theoretically expected associations with relevant outcomes.

## Results

Table 1 summarizes the topics covered in the interviews and online surveys, the data obtained from the participants, and their implications for the program design (illustrative quotes for each identified theme are provided in Supplementary 1, Table S1). These findings were integrated into the final logic model (see Fig. 1) and informed our selection of major determinants of vaping and smoking behavior targeted through our program, included as covariates in our analyses, or examined for gender differences. No single evidence-based intervention was identified that could adequately address all behavioral determinants in the logic model while remaining feasible within the limited number of sessions acceptable for schools and adequately covering vaping. Therefore, we drew on the characteristics of effective previous programs and adapted essential elements into a newly developed intervention. The material review interviews and feasibility testing indicated high acceptability and feasibility of the program, supporting its suitability for evaluation in the main trial.

**Table 1:** Results of interviews and surveys in the development study and feasibility testing and implications for the program design.

Topic	Observation/Learning	Implications
Organizational capacity/ characteristics of the setting	We found a highly diverse student body regarding migration status, spoken languages, and religion (i.e., 60% first/second generation immigrants; 50% reporting a language other than German as most spoken at home; most common religious denominations were Islam (43%) and Christianity (13%) (FEA). Social problems were evident in a number of students’ families, including economic deprivation, family conflicts, drug and alcohol abuse, and lack of parental support for their schooling (STU, STA, FEA). There is a severe shortage of teaching staff, resulting in frequent cancellation of classes (STA, FEA). A large number of students have low levels of reading and German language competence, creating a great need to support students individually (STU, STA, FEA); all students fell further behind the curriculum during school closures in the COVID-19 pandemic (STA). Although smoking prevention is expected to be part of the regular (“framework”) curriculum, it is frequently neglected by teachers due to time constraints (STA). Teachers and governmental coordinators expected that many teachers would not be able to make additional efforts beyond their already demanding everyday responsibilities (STA). They strongly welcomed the external support for smoking prevention as an alternative to teachers being provided with manuals or training to implement it themselves (STA).	Given the extremely demanding conditions in the target setting, any additional burden for school staff needs to be minimized. The prevention program should be conducted by instructors from our study team and limited to a maximum of three sessions to avoid overloading the curriculum.
Vaping/smoking behavior in the target group; salient positive and negative beliefs about being vape- and smoke-free, sources of normative influence, and enablers/barriers	The age at which students begin vaping or smoking varied between schools. In our feasibility testing, lifetime prevalence for vaping/smoking was 11.96% (mean age *M* = 11.74, *SD* = 0.70, 53% female) (FEA). In addition, 14.81% of participants were classified as highly susceptible, reporting they would likely or definitely try smoking or vaping. 35.8% were classified as susceptible, indicating they did not rule out the possibility of trying these behaviors (FEA). Regular use was reported from grades 5–6 onward, although it generally involved only 1–2 students per grade cohort (STU, STA, FEA). Students were frequently exposed to vaping/smoking in various environments, including public spaces, family settings (such as through siblings, parents, and cousins) and near school grounds (such as through other students and teachers). Additionally, they were frequently exposed to online content related to e-cigarettes/vaping, often promotional posts created by influencers on social media (TikTok/Twitch/Snapchat, in particular) (STU, STA, FEA). Key barriers to being vape- and smoke-free identified during the student interviews were: Wishing to belong with a group or be cool, being put under pressure or scared or otherwise bullied, being in trouble/stressed and craving stress relief (STU, STA). Key enablers were knowing about health consequences, effects on fitness and potential addiction, family expectations, associated costs, smell, finding smoking unpleasant, and wishing to belong to a group of friends who do not vape and smoke (STU). Students perceived cigarettes as less attractive due to their odor, cost, and negative health effects (STU, FEA). In contrast, disposable e-cigarettes were often perceived as highly attractive due to their sweet flavors, colorful appearance, low cost, and easy accessibility (STU, FEA). Many students who strongly refused to smoke cigarettes found vapes highly tempting (STU). Although most students had at least some knowledge about the health consequences of smoking traditional cigarettes, there was significant uncertainty and doubt about the potential harm of vaping (STU, FEA).	Prevention efforts should begin as early as fifth grade to reach most students before experimentation and susceptibility become more prevalent. The program should place a strong focus on e-cigarette use and prioritize the cultivation of children’s competencies in the following domains: the ability to discern social influences and resist peer pressure, the capacity for critical thinking and the ability to question information encountered on social media, and the development of adaptive coping mechanisms for stress management and problem-solving. Furthermore, the program should foster positive social norms within the peer group that promote vape- and smoke-free living, while communicating the positive outcomes relevant for the target group.
Group-specific behavioral determinants and needs in prevention	Gender-specific motives to vape or smoke, as reported by students and teachers/pedagogues (STU, STA, FEA): Coping with stress and negative emotions, weight/appetite management (girls); expected status enhancement, such as being cool/belonging/ masculine/impressing girls (boys). Motives to be vape- and smoke-free: avoiding wrinkles and bad smell, disapproval from social environment (girls); fitness/sports motivation (boys). Communication behavior patterns exhibited during discussions about smoking (FEA): Timidity (girls) vs. Boastfulness (boys). SES-specific observations: Students in the less burdened school appeared to have more exposure and knowledge about different smoking/vaping products compared to the lower-SES school with very high strain (STU). Students in both schools reported wanting to stay fit as a motive to be vape- and smoke-free. However, almost all students in the less burdened school reported wanting to perform well in at least one club with regular training sessions, such as playing soccer or hockey, whereas none of the students in the lower-SES school were current members of sports clubs (STU, FEA). Religious beliefs/culture as an important determinant of smoking behavior (STU, STA, FEA): Muslim students referred to smoking/vaping as “harām”/not allowed according to their religion and strictly punished in their families; concluding it is not an option for them; experts reported shaming/bullying of Muslims by their family/friends in response to their smoking; resulting in little consumption or late initiation; water-pipe/hookah being an exception commonly used in many Muslim families.	In order to gain a deeper understanding of group-specific needs in prevention, it is imperative to assess SDOH in students and to explore subgroup effects in the evaluation study. Interventionists should tailor their communication to match the language proficiency of each school class. All intervention materials were designed to use simple language and incorporate diversity in both words and images, e.g., in the choice of illustrated characters and leisure activities. Furthermore, language/terminology used by the students were adopted in intervention materials, e.g., when presenting students with phrases to say when being offered to vape or smoke during the refusal skills training.
Program ideas, materials and experienced lessons in respect to practicability, expected effectiveness, acceptability and feasibility in the setting of structurally burdened schools	Both children and teachers who participated in the material review interviews and feasibility testing provided positive feedback on the program. Students gave high acceptability ratings (*M* = 4.32, *SD* = 0.83; 1 = *did not like the program at all*, 5 = *liked the program very much*), and 94% approved of the program being taught to other students at their school (FEA). Teachers also reported high feasibility (*M* = 4.25, *SD* = 0.83), and 100% said they would approve of using the program in their class again (FEA). However, the teachers perceived obtaining parental consent through our written study information as challenging (STA, FEA). Difficulties in understanding the German language were identified as one main reason.	To simplify the consent process for teachers and to address language barriers, we will provide all information material for parents and students in six languages. Additionally, we will provide teachers with a video introduction to the study (with subtitles) and flyers with concise information.

Sources are coded as follows: STU, STA, FEA.

1. STU, student interviews for needs assessment^54^ (*n* = 33) and material/concept review interviews (*n* = 32) conducted in two lower-SES schools (higher and very high level of structural burden), located in Berlin, Germany. 2. STA,  semi-structured stakeholder interviews for needs assessment and material review/concept (*N* = 7), including five stakeholders who had experienced the workshops or coordinated the program on school level; as well as informal exchange with *N* = 47 stakeholders. 3. FEA, feasibility study which involved online-questionnaires filled out by *N* = 92 students and *N* = 4 teachers accompanying the program; as well as behavioral observation of students during the testing of the intervention.

## Discussion

Our feasibility study found unexpectedly high lifetime prevalence for the age group under study: Approximately 12% across products in a sample of predominantly 11 to 12-year-old students. Compared with data from a German national survey of adolescents aged 12 to 13 years^8^, lifetime cigarette use was similar (3.7%), whereas prevalence estimates for disposable e-cigarettes and hookah were higher in our sample (6.5 and 7.6%, respectively). In addition, 36% of never-smokers in our sample were susceptible to future vaping or smoking. This estimate is higher than the recently reported 23% among 12–13 year olds in the United States^55^, and comparable data for Europe are not available.

In addition to our quantitative results, the qualitative findings also suggest that Grade 6 already represents a critical phase of heightened risk and experimentation; accordingly, we chose to target Grade 5 in our intervention. Our participatory research revealed high exposure to vaping and smoking within families, at school and in public settings. The identified barriers and enablers of vaping and smoking largely mirrored established risk and protective factors of smoking initiation identified in previous research^56^. A notable difference, however, was the particular appeal of disposable vapes: their colorful appearance, perceived pleasant smell, and anticipation of appealing flavors appeared to create a strong temptation that even students committed to not smoking described as difficult to resist. In addition, many students expressed substantial uncertainty about the health consequences of vaping. These findings underscore the need for prevention efforts that explicitly address vaping and equip children with the knowledge and skills needed to resist it.

A central aim of the intervention development was to create a program that serves all schools, with a special focus on addressing the needs of schools serving lower-SES-communities. Extensive participatory research with stakeholders, including governmental- and school-level prevention coordinators, school staff and health interventionists working with the target group allowed us to tailor the intervention more closely to the needs of these settings. Although education reports have pointed to the wider strain on school systems^27^, our stakeholder consultations indicated that this strain also translates into limited capacity to implement vaping and smoking prevention in many schools, especially in those serving lower-SES populations. Key barriers included limited staff capacity, the demands of multilingual classrooms, and curricular backlogs that heighten pressure to prioritize core academic subjects. External support, combined with a relatively brief intervention, therefore appears to be the most feasible strategy for ensuring that students in these schools are reached by prevention efforts. The present intervention was designed with these implementation needs in mind.

### Cluster-randomized controlled trial protocol

The primary objective of the trial is to assess the effects of a school-based prevention program with three training sessions on motivation, smoking, and vaping (primary outcome: intention to be vape- and smoke-free across all modes of use) in children in Grade 5 (Age: 10–11 years) at 3, 6, and 12 months after baseline, compared to an active control group receiving the standard curriculum. Smoking and vaping measures include the use of cigarettes, disposable e-cigarettes, reusable e-cigarettes, hookah, tobacco heater, cigars/cigarillos, and pipe.

We will report intervention content based on the well-established Behavior Change Technique Taxonomy^49^ and examine underlying mediators of the program effects. We will recruit students from schools with elevated sociostructural burden and examine risk and protective factors, intervention effects and mechanisms of action in relation to students’ SDOH. The assessment and analysis of both mediating mechanisms and SDOH as moderators are expected to advance a “precision approach” to prevention – bringing us closer to providing the right preventive intervention to the right person at the right time and in the right place^31,57^.

We hypothesize that children in the intervention group, compared with children in the control group, will exhibit

1. Increased intentions to be vape- and smoke-free (primary endpoint measured at the 12-month follow-up) and
2. Decreased susceptibility to vaping/smoking; vaping/smoking prevalence (lifetime prevalence, 12-month and 30-day prevalence, as well as 30-day-frequency), regular vaping/smoking (≥ once a week), and current vaping/smoking (≥ once a week during the last month).
3. The intervention effects are mediated via
  increased action planning for vaping/smoking refusal; increased vaping/smoking refusal skills (i.e., refusal self-concept and refusal self-efficacy as proxies for actual capabilities); increased self-efficacy to be vape- and smoke-free; increased positive attitudes towards being vape- and smoke-free,
  increased stress and problem coping (i.e., self-efficacy and planning for coping); and heightened intentions to be vape- and smoke-free (for the outcomes of susceptibility and vaping/smoking behavior only); decreased perceived vaping/smoking norms; decreased negative attitudes towards being vape- and smoke-free.
4. We will further test hypotheses about differences in risk and protective factors, as well as in vaping/smoking outcomes, by SDOH subgroups (gender, SES, religion/world view, migration history, spoken language, and health status). A detailed description of this examination can be found in our pre-registration file^37^. Additionally, we will explore differential intervention effectiveness as well as underlying mechanisms of change by subgroup.

### Trial design

**Fig. 2:**
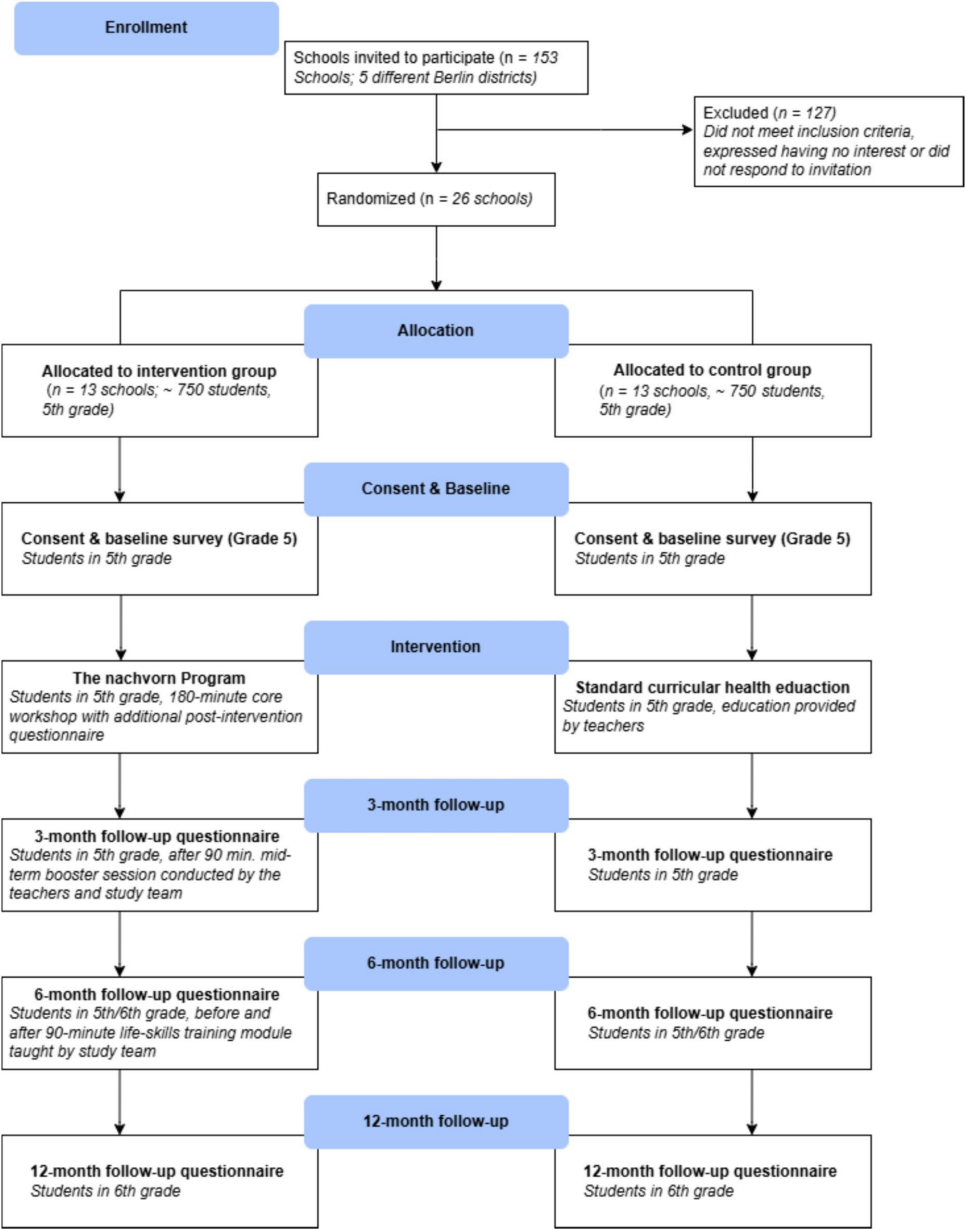
Anticipated participant flow.

A cluster-randomized, controlled, parallel-group, superiority trial will be conducted to investigate the impact of the intervention arm in comparison to an active control arm (standard curriculum taught by the school staff). Intervention lessons and outcome assessments in school will be conducted throughout the school years 2023/24, 2024/25 and 2025/26 in primary schools in Berlin, Germany, that serve Grades 5 and 6 (see Table 2 for the SPIRIT flow diagram schedule of enrolment of schools, interventions, and assessments; see Fig. 2 for the anticipated participant flow). In the German state of Berlin, primary education generally includes Grades 1–6, whereas in most other German states it typically includes Grades 1–4.

**Table 2:** Schedule of enrolment, interventions, and assessments.

Timepoint	Study period								
	Enrolment	Allocation	Post-allocation						Close-out
	-t1	0	t0	t1^b^	t2	t3	t4	tn	tn
Enrolment:									
Eligibility screen	X								
Informed consent	X								
Allocation		X							
Interventions:									
Prevention program									
Standard curriculum (active control)									
Assessments:									
Outcomes: intention, susceptibility			X	X	X	X	X	X	X
Outcomes: vaping and smoking behavior			X		X	X	X	X	X
Proposed mechanisms / mediators^a^			X	X	X	X	X	X	X
Program evaluation: students and teachers^b^				X	X	X^c^			
Baseline covariates			X						

T0: Baseline, approximately one week or immediately before the first core workshop, T1: immediately after core workshops 1 and 2, T2: 3 months post-intervention, T3: 6 months post-intervention, T4: 12 months post-intervention, Tn = optional follow-up surveys up to the age of 21. ^a^The following measures are only assessed at T4–T^n^: Affective experience and anxiety (intervention group only), stress and problem coping self-efficacy/planning (intervention and control group). ^b^ measured only in the intervention arm. ^c^  measured in students only.

The detailed research plan has been registered with the Open Science Framework (25/11/2023)^37^, before the start of baseline data collection, and later with the German Clinical Trials Register, DRKS00036358; 06/03/2025), while recruitment was ongoing. At the time of manuscript submission, recruitment and baseline collection were ongoing. This study protocol and subsequent reports are prepared following the SPIRIT 2013 and CONSORT 2010 statements^58,59^.

### Study population and eligibility criteria

The study will be conducted in the German state and capital of Berlin, an urban area with a smoking prevalence and tobacco-related mortality ranging among the highest of the German states [^76^]. Eligible schools must be located in one of six Berlin districts selected a priori because they rank among the highest in the city in the proportion of students exempt from extra payments for learning materials and/or entitled to the Education and Participation Pass (Bildungs- und Teilhabepaket, BuT) [^77^] These districts are Mitte, Neukölln, Spandau, Reinickendorf, Lichtenberg, and Friedrichshain-Kreuzberg.

To be eligible, schools must be primary or secondary schools that teach both Grades 5 and 6 or, if they normally teach mixed-grade classes, must agree to form grade-specific groups for the duration of the prevention lessons. Although the intervention itself targets Grade 5 students, participating schools also had to teach Grade 6. This requirement was intended to facilitate the 12-month follow-up and reduce attrition due to school transitions between Grades 6 and 7.

Principals will be required to provide written consent to participate in the study, including agreement not to involve any other external vaping or smoking prevention provider, or at least to inform the study team if they do so. In addition, for a school to be included in the study, all classes in the target grade will be required to participate. At the student level, both parents and children will be required to sign consent forms for the data assessment.

### Recruitment procedures

We will use a variety of contact methods in our recruitment efforts. Prior to our direct contact with the schools, the prevention officers at the Berlin Senate Department for Education, Youth and Family (SenBJF) will promote the study through a program flyer and an informational e-mail to the target schools. We will gradually contact all primary school secretaries in the selected districts directly by phone to reiterate the offer. Whenever possible, we will request a direct phone call with the school principals to inform them about the program.

In all of these cases, where schools have expressed initial interest, we will arrange in-person recruitment meetings at the schools with principals. We will provide them with a comprehensive presentation of the lesson content, the study itself, and the data assessment, and may use the opportunity to schedule classroom visits.

### Informed consent

Prior to the initial classroom visit, the study team will be available to attend parent meetings and to personally explain all important details of the study. Alternatively, teachers will be instructed to provide parents and children with verbal and written information material about the study. This material includes a comprehensive information sheet, a video, and a flyer summarizing key points for informed consent, provided in English, German, Arabic, Turkish, Russian, and Ukrainian. In the event that students, parents, or teachers require specific clarification, the study coordinators will be available via telephone and email. Prior to the baseline assessment, teachers will collect all consent forms and check them off on a class list for oversight. Before administering the survey on site, the study team will provide a verbal summary of key study information, emphasizing anonymity, the voluntary nature of participation, and the right to withdraw. The team will then review consent forms to begin the assessment. The consent documents feature various levels of consent: Consent to participate in the survey will be a prerequisite for enrollment, while consent to be photographed in the photo challenge and consent to be interviewed after the workshops will be optional.

### Allocation and blinding

After the school principals have given their written consent to participate, schools will be randomly allocated to intervention and control groups by the study coordinator with no role in the analyses of study outcomes. We chose to randomize at the school level to avoid potential contamination effects between intervention and control classes, i.e., through student interaction. During recruitment, blocks of newly enrolled schools (varying in size, with a minimum of 2, stratified by school SES [Berlin school classification index]^37,50^ and number of classes) will be created and allocated sequentially to intervention and control groups in a 1:1 ratio. To remove any researcher involvement, we will use an automated randomization process in R based on a list of random seeds generated prior to school enrollment. Moreover, the data analyses of study outcomes will be conducted by an external statistician unaware of the coding of treatment groups.

School personnel and research staff involved in organizing and or conducting workshops and assessments will inevitably be aware of the treatment condition (i.e., due to visible changes to the classroom environment during the intervention). To mitigate researcher influence, we will enforce standardized and comparable interactions between researchers and students during outcome assessments via meticulously crafted procedural scripts and rigorous training of study personnel. For participant blinding, both control and treatment groups will receive almost identical briefings regarding the study objective, i.e., enhancing the understanding of nicotine use in children, with no mention of the planned comparison of group outcomes.

### Intervention and comparator

The intervention is taught during school hours by a tandem of research assistants with an expertise in human medicine, health sciences or psychology (i.e., (under-)graduate students or professionals), whose members alternate in leading the intervention (i.e., the psychologist discusses mental health and addiction; the medical practitioner explains biological processes).

The intervention program comprises three basic components that are conducted at three-month intervals in the course of one school year:

1. A 180-min *Core Workshop* delivered by the study team that can be taught in two separate sessions. The primary objective of this workshop is to educate students about the consequences of vaping and smoking, enabling them to identify and resist unfavorable social influences and to cultivate positive social norms. Additionally, the workshop aims to facilitate the acquisition of critical thinking about the influences of advertising and social media.
2. A 90-min mid-term *Booster Session*, which serves to refresh and deepen the content of the core workshop. The booster consists of two parts: approximately 45 min taught by the class teacher and 45 min taught by the study team. The teacher-led part consists primarily of the students creating a poster advertisement for a vape- and smoke-free life, a task that can be implemented with minimal teacher preparation and no additional training. Materials are provided to schools in advance, and the task serves as preparation for the subsequent visit by the study team.
3. A 90-min, more comprehensive *Life Skills Training* module, led by the study team, focusing on problem solving and managing uncomfortable emotions, such as stress and anxiety.

An overview of intervention content, employed BCTs and targeted theoretical determinants is provided in Table 3.

**Table 3:** Overview of workshop modules, key content, BCTs and targeted theoretical determinant of vaping and smoking behavior.

Workshop	Content/key message	Key BCTs^a^	Key change objective in our assessment model^a^
Common techniques and objectives in Core Workshop and Booster		Information about antecedents [4.2], Information about health consequences [5.1], Salience of consequences [5.2], Information about social and environmental consequences [5.3], Credible source [9.1], Social reward [10.4], Framing/reframing [13.2], (Autonomy-supportive interaction style, not in taxonomy [^65^])	Instrumental/Experiential attitude Intention Refusal self-concept/ Self-efficacy to refuse/ Self-efficacy to be vape- and smoke-free Descriptive/Injunctive norm
Core Workshop Part I	*Future aspirations:* Sharing future goals; instructors frame vape- and smoke-free living as an important decision for the students’ future. Health: Expert quiz covering cardiovascular system functions; harmful ingredients and health consequences; effects on appearance (partly adapted from [^68^), general fitness and athletic performance; smoking experiment demonstrating tar deposition in the lungs [^69^]	Comparative imagining of future outcomes [9.3], Imaginary reward [16.2]	
	*Social influences and refusal training:* Sharing experiences with peer pressure; strategies to refuse smoking; action planning using a volitional help sheet (adapted from [^70^])]; model dialogue by the instructors; role-play exercise of saying ‘no’	Problem solving [1.2], Action planning [1.4], Commitment [1.9], Instruction on how to perform a behavior [4.1], Demonstration of behavior [6.1], Behavioral practice/rehearsal [8.1], Verbal persuasion about capability [15.1], Prompts/cues [7.1]	Action planning
Core Workshop Part II	*Addiction and well-being:* Understanding addiction development including the role of social influences; implications for mental well-being. *Monetary costs:* Exploring the financial impact of smoking versus being vape- and smoke-free. *Marketing and Lobbyism:* Examining tobacco industry influence on tobacco control-policies; manipulative marketing strategies; ethics in advertising	Information about emotional consequences [5.6], Information about health consequences [5.1], Information about others’ approval [6.3], Pros and cons [9.2], Imaginary reward [16.2]	
	*Living vape- and smoke-free:* Normative feedback on the high percentage of vape- and smoke-free students in the class (adapted from [^71^]); Danish campaign presentation: “cool without smoke” [^72^] ; video message from German actor introducing the “photo challenge”: taking pictures for an advertising campaign demonstrating the appeal of a vape- and smoke-free life	Feedback on behavior [2.2], Information about others’ approval [6.3], Social comparison [6.2], Identification of self as role model [13.1], Identity associated with changed behavior [13.5], Reward incompatible behavior [14.7], Prompts/cues [7.1]	
Booster Sessions (90 min led by class teacher, 45 min led by study team)	*Recap and sharing:* Reflection on the content of previous workshops; sharing experiences with refusing smoking and recapitulating strategies using the volitional helpsheet (adapted from [^70^]). *Poster creation and presentation:* Creating and presenting advertisement posters around the theme of being vape- and smoke-free, using photos taken in the Core Workshop Part II	Problem solving [1.2], Social support [3.1], Feedback on behavior [2.2], Social comparison [6.2], Identification of self as role model [13.1], Identity associated with changed behavior [13.5], Reward incompatible behavior [14.7], Action planning [1.4], Commitment [1.9]	Action planning
Life Skills Training^a^	*Resources:* Recognizing personal resources and committing to daily self-care; “Emergency-Powercard” with 24/7 support hotlines. *Coping and problem solving:* Action planning for challenging situations and unpleasant emotions; breathing exercise (adapted from [^73^]	Reduce negative emotions [11.2], Problem solving [1.2], Instruction on how to perform a behavior [4.1], Demonstration of behavior [6.1], Behavioral practice/rehearsal [8.1], Action planning [1.4], Commitment [1.9], Prompts/cues [7.1]	Coping with stress Distress Action planning

^a^The objective of the life skills training is to cultivate adaptive strategies for coping with stress and problem solving. The interventionists do not mention smoking and vaping; therefore, in the corresponding row of the table, we provide codes for BCTs aimed at fostering adaptive coping with stress and problem solving. With regard to the intervention’s overall goal of students being vape-and smoke-free, the entire session can be coded as the BCT “Reduce negative emotions [11.2]”.

### Theoretical foundation and key intervention characteristics

The program theory (logic model; Fig. 1) was developed following the Intervention Mapping approach^39^ (see Development study) and is primarily based on the Integrated Behavioral Model^40^, rooted in the Reasoned Action Approach^51^. Accordingly, the evaluation focuses on intention to be vape- and smoke-free as the most proximal determinant of behavior, as well as on its key determinants, including attitudes, perceived norms, and personal agency. The model was extended to include three additional constructs assumed to be particularly important intervention mechanisms, namely action planning^45^, refusal self-efficacy [^60^] and refusal self-concept (developed within this study [^61^]). Refusal-related constructs have, alongside perceived norms, emerged as the most consistent mediators of effects in previous substance use prevention research [^62,63^]^64^, while action planning is assumed to support the translation of intention into behavior^45^. In addition, the model incorporates more distal factors, such as life skills [^64^]  (i.e., stress coping and self-esteem), based on prior research^56^ and the findings of our development study.

The program is introduced under the name *nachvorn* (ahead) and is based on three main principles. First, the intervention emphasizes positively framed messages on the benefits of being vape- and smoke-free for students’ overall well-being. Second, the interventionists use an autonomy-supportive interaction style, which is meant to foster students’ experience of autonomy, relatedness and competence, thus strengthening their sense of self-determined motivation to be vape- and smoke-free [^65^]. This interaction style is operationalized through specific communication behaviors and interactive teaching methods, all of which have been demonstrated to be related to a heightened sense of students’ autonomy [^65^]. Specifically, interventionists a) allow time for students to talk and share their experiences, use reflective listening and empathically acknowledge students’ perspectives, b) are highly responsive to student-generated questions c) support active learning and independent work, and d) provide positive feedback and encouragement during knowledge and skills acquisition. Consistent with this approach, interventionists emphasize students’ freedom of choice and their capacity to adhere to their own decisions, even in the face of difficulties. Interactive learning methods include an “expert quiz,” demonstrations by interventionists and by role models via video message, role play, group discussion, and creative group work. Together, these strategies are intended to support students’ sense of autonomy.

When educating students about marketing strategies for tobacco products (including the design of highly appealing vapes and the targeting of children), interventionists use a *value-alignment approach* to motivation [^66^], aligning the act of being vape- and smoke-free with commonly shared values in the target age group (i.e., the strong desires for autonomy and social justice). The target behavior is thus framed as a way for students to assert their autonomy from the manipulative tobacco industry and to stand up to its immoral methods. Third, the program places significant emphasis on the similarities between smoking and vaping throughout its duration. The term “smoking” is employed as an umbrella term for the inhalation of smoke or vapor that may affect physical and mental health.

### Active control condition

In the active control arm, school staff will teach 5th grade students the standard curriculum, which is supposed to cover the topics of addiction and tobacco use prevention [^67^]. The study team will visit the control classes for data collection, with comparable but slightly less contact time than in the intervention group classes. In an effort to keep study participation desirable for schools assigned to the control arm, we will offer to teach intervention workshops to a different cohort of students (i.e., those in grades 4 or 6, or students in grade 5 the following school year).

### Strategies for enhancing and monitoring intervention fidelity

We developed the process evaluation and fidelity plan based on established guidelines ^74,75^. An intervention manual and scripted curriculum will be used to ensure interventionists’ understanding of the logic model and active treatment components and to standardize interaction with students. The material further covers background knowledge on health consequences, and proposes responses to frequently asked participant questions and typical challenging situations in class. Finally, it defines rules for dealing with time constraints and adapting the intervention to the needs of the classes while making sure to deliver the core intervention components. Interventionists will receive additional training during staff workshops and peer-to-peer coaching, including role playing the classroom interactions with opportunities to discuss the Behavior Change Techniques / Autonomy-Supportive Interaction with the intervention development lead. Following each intervention session, interventionists will fill out a post-intervention questionnaire and assessment log, which may serve as a reminder of the intervention components, active ingredients and interaction style to be delivered [^74^]. Finally, weekly staff meetings with the intervention development lead will be held for the purpose of clarifying questions and exchanging experiences regarding recently taught interventions and any deviations from protocol.

### Analytic sample and sample size

We calculated the sample size needed to detect a difference between intervention and control group at 80% power (*α* = 5%) in accordance with the National Institutes of Health recommendations for the analysis of cluster-randomized trials^78^. Consistent with randomization at the school level, the school was treated as the primary clustering unit in a two level structure (school- and student-level observations).

The following estimates of the relevant factors were derived from previous research: Focusing on our continuous primary outcome variable intention, we conservatively assume an intervention effect size of Cohen’s *d* = 0.2^79^. Based on the current literature, the intraclass correlation (ICC) of this psychosocial variable is expected to be lower than that of overtly visible risk behaviors (e.g., smoking) that may be subject to social mimicry^80^. We therefore used an ICC estimate of 0.005, as reported by Siddiqui et. al^81^ for the intention to smoke in 47 schools. Assuming an average cluster size of 60 students with usable data per school, based on an average of 3–4 classes per school with 22 students per class [^77^], our analysis yields a minimum sample size of 10 schools in each study arm (1,200 students). Expecting an attrition rate of up to 20%^52^, we aim to recruit *N* = 1,500 students in at least 26 schools (see Fig. 2: Anticipated participant flow). Because the ICC assumption is subject to uncertainty and higher ICC values would reduce effective power, we will recruit beyond this minimum target if feasible.

### Data collection

From baseline to 12-month follow-up (i.e., T0–T4), all measurements will take place during school hours and will be administered using tablets that present online questionnaires (paper-based questionnaires may be used in special cases). At baseline only, students will complete a worksheet with sample items to familiarize themselves with the question format and survey-specific terminology. The worksheet explicitly introduces “smoking” as an umbrella term for the use of any tobacco or vaping product and “smoke-free” as abstinence from all such products. To promote a shared understanding across schools, these terms are illustrated using visual mind maps (i.e., one mind map presenting “smoking” together with images of all product types assessed in the study, and one presenting “smoke-free” together with the same product images with a red cross over them). Example items are then completed with these definitions in mind, supported by brief instructor guidance. In addition, students will generate an identification code and enter it into the online questionnaires at each assessment, thus enabling the matching of data across waves without the collection of other personally identifying information. Students will then be asked to complete the questionnaire in silence. Assessors from our research team are familiar with the questions and operationalized constructs and will be trained to explain any questions that may arise for individual students. In addition to the collection of quantitative data, semi-structured individual interviews (lasting approximately 60 min) will be conducted with approximately 10 students after their participation in the core workshops.

### Measures

Table 4 presents a summary of the measurements utilized in the student questionnaires at each assessment time-point. For a comprehensive overview of the measurements employed in the student, teacher, and interventionist surveys, including construct order, number of items, translated example items, and source references, see Supplementary 2, Tables S2–S5. Most constructs are assessed using brief two- to three-item scales, with a small number of single-item measures to reduce respondent burden for the target group. Key proposed mechanisms are measured using well established operationalizations from the Reasoned Action Approach and related research^51^. A small number of measures were drawn from previous studies (see Supplementary 2) based on their psychometric properties. Other scales assessing mechanisms and covariates were developed within the project. In our feasibility sample, internal consistency for all multi-item scales used in the student questionnaire was acceptable (Cronbach’s α = 0.72–0.83).

**Table 4:** Summary of measurements used in the student questionnaires.

Measurement	T0	T1^a^	T2	T3	T4	Tn
*Outcomes*						
Intention to be vape- and smoke-free^b^, susceptibility to vaping and smoking	X	X	X	X	X	X
Lifetime prevalence, 12-month and 30-day prevalence, current vaping/smoking, regular vaping/smoking, 30-day frequency	X		X	X	X	X
*Proposed mechanisms / mediators*						
Personal agency: self-efficacy to be vape- and smoke-free, refusal self-efficacy, refusal self-concept	X	X	X	X	X	X
Action planning	X	X	X	X	X	X
Descriptive and injunctive norms towards being vape- and smoke-free	X	X	X	X	X	X
Attitude towards being vape- and smoke-free (direct measure)	X	X	X	X	X	X
Outcome expectations (indirect attitude measure)	X	X	X	X	X	
Stress and problem coping self-efficacy, stress and problem coping planning, affect^a^, anxiety^a^				XX	X	X
*Program evaluation*						
Satisfaction		X	X	X		
Careful responding /attention	X	X	X	X	X	X
*Sociodemographics and covariates*						
Age, gender	X	X	X	X	X	X
SDOH, country of origin, objective SES, subjective SES, ethnicity/racial identity, language, religion, general health	X					
Vaping/smoking of relevant others, parental disapproval of vaping/smoking, accessibility, school attachment, self-esteem, satisfaction with looks, distress	X					

T0: baseline, conducted approximately one week or immediately before the first core workshop, T1: immediately after core workshops 1 and 2, T2: 3 months post-intervention, after booster session, T3: 6 months post-intervention, after life skills training, T4: 12 months post-intervention (assessment only), Tn = optional follow-up surveys up to the age of 21. “XX” indicates assessments conducted both immediately before and after the workshop in the treatment condition (T3).

^a^ measured only of the intervention arm participants.

^b^ intention will be modelled as an additional mediator of intervention effects on behavioral outcomes, alongside the other proposed mechanisms (e.g., self-efficacy, refusal self-concept, action planning).

Given the linguistic and educational diversity of the study population, these questionnaires were phrased using simplified wording and grammar. Assessors will be trained to develop a shared understanding of questionnaire constructs and item meanings in order to support standardized clarifications during data collection. Recurring clarification needs, particularly during baseline assessments, are discussed in weekly staff meetings and incorporated into the assessment script to further standardize assessor support across schools.

*Primary endpoint (T4).* The primary endpoint for this study is the difference in students’ *intention to be vape- and smoke-free* (i.e., to abstain from using any vaping or tobacco product) between intervention and control groups (primary endpoint measured at T4, 12 months post-intervention). This endpoint will be measured by two items on a 5-Point-Likert-Scale, i. e.: “I want to be smoke-free (that is, not to smoke) for the next six months” (1 = *strongly disagree*, 5 = *fully agree*)^51^).

*Secondary behavioral endpoints.* *Susceptibility to vaping or smoking*, capturing the absence of a strong commitment not to vape and smoke^82^, will be assessed based on items used in previous research^83,84^ and coded binarily (0 = *Committed non-vapers and non-smokers*, 1 = *Susceptible to vape or smoke*). Students will be asked to report their expected behavior in three scenarios: “Do you think you will try smoking in the future?”, “If one of your closest friends offered you something to smoke, would you smoke it?”, “Do you think you will smoke at some point within the next year?”, using a 4-Point-Likert scale (1 = *definitely not*, 4 = *definitely*). Students will be considered committed non-vapers and non-smokers (vs. susceptible to vaping or smoking) only if they report “definitely not” on all questions. This dichotomization is consistent with the established susceptibility index literature showing strong prospective predictive validity of the binary measure for initiation of cigarette and e-cigarette use^82,85^. Alternative codings, including categorical and continuous versions, will be explored in secondary/sensitivity analyses as specified in the preregistration^37^.

*Lifetime, 12-month, and 30-day prevalence of vaping and smoking* (binary coding), *30-day-frequency, current use* (binary coding; any current consumption, including only occasional use during the last month), and *regular use* (binary coding; vaping or smoking more than once a week during the last month), will be assessed using adjusted measures from the KiGGS (German Health Interview and Examination Survey for Children and Adolescents, Wave 1)^86,87^*.* Prevalence and frequency measures will be collected separately for individual product categories (i.e., cigarettes, disposable e-cigarettes, reusable/refillable e-cigarettes, hookah, tobacco heater, cigars/ cigarillos, pipe) and will be aggregated to calculate prevalence/frequencies across products for the main analysis.

*Proposed mechanisms (mediators).* As specified in our logic model, the main proposed determinants of behavior and mechanisms of change to be underlying the intervention effects on the primary outcome (intention to be vape- and smoke-free) include self-efficacy to be vape- and smoke-free, refusal self-concept, refusal self-efficacy, action planning, descriptive and injunctive norms towards being vape- and smoke-free, and attitudes towards being vape- and smoke-free (direct measure). For behavioral outcomes (i.e., susceptibility, prevalence, and frequency), intention will be modelled as an additional mediator alongside the mentioned psychosocial determinants (e.g., self-efficacy, refusal self-concept, action planning). In addition to the quantitative assessment of mediators via student questionnaires, the proposed mechanisms of change will be explored qualitatively in semi-structured interviews conducted after the core workshop.

*Additional measures.* The baseline assessment will include a number of sociodemographic variables and proposed determinants of vaping and smoking. Among these, age, gender, objective SES, school attachment, parental vaping/smoking, best friend’s vaping/smoking, self-esteem and distress will serve as covariates in the main analysis of intervention effects on the primary outcome. In addition, we will assess SDOH to examine differential intervention efficacy and underlying mechanisms of action by SDOH subgroup. For the purpose of sensitivity analyses, the student questionnaires will also include different attention screeners (e.g., “Please show us that you are reading the questions carefully and mark response option ‘Definitely’; “How much do you agree? ‘I am currently responding to a questionnaire’) and an item to detect careless responding^88^.

*Fidelity, acceptability, reach.* We will examine *reach* using descriptive, quantitative information on rates of school and of student consent and participation in different study waves (see^37^ for a detailed list of indicators). *Fidelity* data will be obtained from teacher (after teaching the teacher booster session at T2) and interventionist self-reports (after teaching at T1, T2, and T3) on the quality of their intervention delivery. Ratings will include measures adapted from previous studies, i.e., the *overall quality* and quality of different parts of the workshops [^70^*]*, as well as the study team’s self-reported *competence in program delivery*^89^ and autonomy-supportive interaction style [^65^]  (e.g., use of appropriate language, adjusting the pace to students’ needs, reflective listening, empathic paraphrasing, building on students’ contributions, and actively engaging students), as well as *dose* (rate of intervention sessions taught as planned; duration of each session; percentage of intended content delivered per part; as well as substantive deviations from the intervention protocol).

*Acceptability* measures are collected from teachers observing the core workshop (T1) as well as from students and interventionists after the core workshop (T1), the study team’s booster session (T2) and the life skills training (T3). These include teacher- and student-reported satisfaction with the program^52^ and positive and negative events reported by teachers and interventionists.

### Data monitoring

There will be no Data Monitoring Committee or interim analyses with respect to the study outcome. In order to determine the appropriate point at which to cease recruitment, members of the study team will continuously monitor the acquired sample size including only data from students who provided apparently valid self-generated pseudonyms (i.e., combinations of numbers and letters in the expected sequence).

### Assessment and monitoring of harmful effects

In addition, the intervention development lead will monitor the occurrence of any adverse events that may result in student harm and may need to be managed appropriately. However, we have used the numerous teacher interviews and feasibility tests to anticipate a number of possible adverse events that may occur (i.e., bullying during role-playing or group discussions) and to include guidance on how to appropriately manage these scenarios in our training for interventionists.

### Analysis

The analyses on intervention effects will be conducted on an intention-to-treat basis, including data from all students based on the groups they were randomly assigned to. The main confirmatory analysis pertains to the group difference between intervention and control group in intention to be vape- and smokefree at the 12-month follow-up (T4), adjusted for baseline (T0). All other analyses, including analyses of secondary behavioral outcomes and additional time points, will be considered secondary or exploratory, and no formal multiplicity adjustment will be applied.

Group differences in secondary behavioral endpoints (i.e., prevalence and frequency measures) will be analyzed across all product categories (e.g., prevalence defined by the use of at least one of the products presented in the questionnaire) as well as individually for each product reported by at least 1% of the sample.

For continuous and dichotomous outcomes, we will use generalized mixed effect regression analyses (i.e., linear regression for the outcome of intention to be vape- and smoke-free; logistic regression for [binary] susceptibility, prevalence measures, current vaping/smoking and regular vaping/smoking). The primary analyses will use a two-level structure, with students at Level 1 nested within schools at Level 2, reflecting the school-level randomization and the role of the school as the primary clustering unit. In regard to count data, we will use Generalized Estimating Equations to conduct zero-inflated negative binomial regressions for count outcomes with many zeros, i.e., the 30-day frequency. We will investigate intervention effects using the criterion of statistical significance (*p* < 0.05) of the regression coefficients for the treatment condition variable (adjusting for prespecified baseline covariates; all measured at T0: intention, age, gender, parental vaping/smoking, best friend’s vaping/smoking, objective socioeconomic status, school attachment, self-esteem and distress). All models will be specified with a maximal random effects structure and progressively simplified as needed to reach convergence.

To handle missing data appropriately, we will conduct a comprehensive analysis of attrition patterns, multiple imputation and sensitivity analyses to check the robustness of our inferences to departures from the underlying assumption of missingness at random.

We will initially test the hypothesized mediators of intervention effects in simple mediator models before proceeding with a multiple mediation analysis. All mediation analyses will account for the nesting of students within schools using mixed-effects modelling approaches.

*Program evaluation by social determinants of health.* We will investigate SDOH subgroup differences in absolute levels of risk factors and outcome variables, as well as differential predictive power of those risk factors for intention, susceptibility, and vaping/smoking. Last, we will explore differential intervention effects and mediators by reporting effect sizes by subgroup.

*Further examination of results.* In additional secondary and sensitivity analyses, we will examine alternative modelling strategies that incorporate class-level effects, the use of alternative indicators or variable codings, and longitudinal models based on all available measurement time points. The quantitative analyses described above will be conducted using RStudio and SPSS. Further details of the statistical analysis plan are available in the pre-registration^37^.

### Confidentiality and dissemination

The data protection concept has been developed in accordance with the Berlin Data Protection Act, the German Federal Data Protection Act and the EU General Data Protection Regulation and has been reviewed by the data protection team of the Charité Clinical Trial Office. To link data across surveys, we will only use pseudonyms generated by the participants, and we will not collect any directly identifying information such as names or personal addresses. Pseudonyms will include codes that replace school names. We will store participant data and a list linking school codes to school names separately on password-protected Charité databases accessible only to members of the study team or, in the latter case, to the study coordinators. Paper-based records of participant data, such as questionnaires or informed consent forms for adult participants, will be stored in locked filing cabinets at the study site. Student and parent consent forms, as well as a list of participating students per class, will be kept securely by school personnel and given to the study team for review on site before each assessment. We will ask schools to destroy these documents at the end of the study. We may publish trial results in a variety of forms, including journal articles, academic theses, and conference or media presentations.

## Discussion

In this paper, we present a theory- and evidence-based vaping and smoking prevention program resulting from intervention mapping, as well as a protocol for testing its effects in a cluster-randomized controlled trial. Extensive stakeholder engagement enabled the development of a feasible and acceptable intervention that was well received in the feasibility testing. Three interactive workshops taught by graduate students with expertise in medicine and psychology were designed to motivate students to live a vape- and smoke-free life and foster intra- and interpersonal skills necessary to achieve this goal.

The planned cluster-randomized controlled trial is one of the first to evaluate a vaping prevention program using a rigorous research design that reflects the state of the art in behavior change science. Systematically testing mediating mechanisms of change can disseminate future intervention design and enable economic solutions for school-based prevention. However, it must be acknowledged that the present intervention is a complex one, involving multiple BCTs, which limits definitive conclusions about the effect of any single BCT independent of the other techniques used. A viable next step is to complement intervention testing in real-world settings with controlled experiments that allow testing of individual BCTs in isolation^90^.

We will recruit an understudied, diverse sample of students whose high risk of vaping and smoking makes prevention imperative. The baseline survey will collect comprehensive data on the students’ backgrounds, allowing for the examination of differential needs and intervention effects related to students’ SDOH. The study’s findings have the potential to advance precision prevention, thereby leading to improvements in health for all through more personalized intervention approaches. Additionally, the investigation of heterogeneity by SDOH subgroups may offer insights into the underlying structural causes of disparities in vaping and smoking and may inform complementary policy approaches for their reduction. Given the inherent limitations in statistical power when analyzing small subgroups, we consider the planned analyses exploratory in nature; nevertheless, the results have the potential to generate hypotheses on subgroup differences and subsequently facilitate their confirmation in future meta-analyses.

Randomization at the school level was chosen to reduce the risk of contamination, as all classes within a grade cohort at a school either received the intervention or served as controls. Nevertheless, some residual contamination cannot be excluded, for example via peers, siblings, or social media exposure across schools and grades. In addition, while participating schools were asked not to implement other external vaping/smoking prevention activities during the study period, or to inform the study team if they did so, some influence of additional prevention activities cannot be fully ruled out in a real-world school setting. If such activities are reported, we will describe them transparently and consider their potential impact in sensitivity analyses.

*Further limitations concern sampling and measurement.* In structurally burdened school contexts, parental consent may make study inclusion more difficult and introduce selection bias if students whose parents do not provide consent are systematically underrepresented. This challenge was already identified during the development study and informed efforts to reduce participation barriers, including adapted study information materials, translated video materials, and close collaboration with schools. These efforts will be continued throughout the trial. Finally, relying on self-report measures for all variables is a study limitation due to the potential for bias; however, given the context of burdened schools, we gave priority to feasibility and economy in the study and intervention procedures.

## Supplementary Information

Supplementary Information 1.

Supplementary Information 2.

## Data Availability

De-identified data and analytic code will be published via in the OSF repositories upon completion of the study. Consent forms are already available at [https://osf.io/2zvht/].
