# Supplementary material for: Development of a school program for vaping and smoking prevention and protocol for a cluster randomized controlled trial in fifth grade students
Source: Sci Rep. 2026 May 7;16:13263. doi: 10.1038/s41598-026-45720-w (PMC13153207; doi:10.1038/s41598-026-45720-w)
Supplement: Supplementary file 1 — Supplementary Information 1. [file 41598_2026_45720_MOESM1_ESM.docx]

**Supplementary File 1: Development Study**

**Supplementary Note S1:**

**Detailed procedure for semi-structured interviews and stakeholder exchanges**

**Student interviews (*n* = 33).** At the beginning of each interview, the interviewer presented a graphical depiction of various nicotine products (cigarettes, refillable e-cigarettes, disposable e-cigarettes, and hookah) along with illustrations of individuals using them. Students were first asked about their knowledge of these products. The interviewer then briefly explained the differences between them and introduced the term “smoking” as an umbrella term for all depicted products. The interview subsequently assessed students’ experiences with nicotine consumption in their social environment, including whether they had observed others using these products. Finally, students were asked about their own experiences with nicotine use.

The main part of the interview employed a *Salient* *Belief Elicitation Approach* structured according to the Integrated Behavioral Model (IBM) / Reasoned Action Approach (RAA) [1–3]. According to these frameworks, behavior is ultimately determined by salient- or “top of mind” - beliefs about performing the behavior. To identify salient beliefs about vaping and smoking common in the target group, students were asked to think about being vape- and smoke-free (i.e., not using any nicotine or tobacco product) for the six months ahead, and then to describe expected affective responses (“How do you imagine would you feel?”) and expected positive and negative outcomes (“What are the advantages? What are the disadvantages?”), normative beliefs on other’s behavior (“Who of the people you are close to is smoking?”) and other’s expectations (“Who would like you being smoke-free? Who would not like this?”), and efficacy and control beliefs (“Imagine you wanted to be smoke-free for the next six month. How certain are you you could?” and „What things make it easy for you? What things make it hard for you?“). Additional questions focussed on control beliefs about accessing nicotine products (“If you wanted to smoke, how easy would it be for you to get any of those products? Why?”), a deeper exploration of perceived peer pressure (“Do you think that someone might try to persuade you to smoke or put you under pressure to do so?“), self-efficacy to refuse offers (“Imagine someone offers you a cigarette. How certain are you could say ‘no’? …and if it’s a vape? …hookah?”). In addition to the typical approach used in belief elicitation studies, this study invited students to elaborate on perceived gender differences in nicotine consumption, as well as differences in drivers of initiation and perceived advantages of regular use. Additional questions examined school policies concerning teachers’ and students’ nicotine and tobacco use on school grounds, students’ social media use and their current social media role models, as well as students’ exposure to nicotine and tobacco product advertising and their understanding of common advertising strategies.

**Student review of material and concept (*n* = 29 of *N* = 62)**. Fifteen students participated in a simulated intervention format in which the interviewer delivered a condensed version of the workshop to one or two students, using draft presentation slides, worksheets and other materials. The remaining fourteen students took part in post-lesson material review interviews after participating in feasibility testing of the workshop during regular class time. In these interviews, the materials were re-presented as a visual reminder of the experienced program. In both formats, sessions alternated between brief presentation or review of intervention content and think-aloud prompts. When students did not spontaneously verbalize their thoughts, the interviewer used open-ended prompts (e.g., “What are you thinking right now?”), asked them to describe what they saw, or posed targeted comprehension questions.

Interview foci were iteratively adapted to emerging feedback needs. For example, ten students were interviewed in pairs rather than individually to actively test and provide feedback on a role-play exercise (e.g., practicing refusal skills in peer-pressure situations).

**Stakeholer interviews and exchanges.** Stakeholder and expert interviews and exchanges included both needs assessment and material and concept review components.

The needs assessment part had a focus on schools’ organizational capacity and teacher’s capacity to conduct or support prevention efforts. Further questions mirrored those in the students’ needs assessment but addressed students’ vaping and smoking behavior and its determinants, gender differences and group-specific needs from an external perspective. For example, teachers were asked about students’ vaping and smoking behavior; their expectations regarding consequences; perceived norms and expectations communicated by students’ significant others; and barriers and facilitators related to students being vape- and smoke-free.

Material and concept review, as well as informal exchanges, included feedback prompts either following a presentation of the intervention concept by the interviewer (i.e., planned content, duration, and intervals between sessions; done in semi-structured interviews with *n* = 2 stakeholders, informal exchanges with *n* = 42 participants) or direct experience with the draft intervention in the feasibility study (*n* = 5 semi-structured interviews conducted post-lesson). Initial feedback was elicited through an open-ended question designed to capture spontaneous impressions. This was followed by targeted questions focused on the feasibility of the proposed or experienced program within the intended setting. Depending on the evolving needs of the intervention development process, the interviewer subsequently posed specific design-related questions or invited think-aloud feedback on particular content elements or draft materials. An exception to this procedure were informal exchanges with five individuals (*n* = 5) who were observed during their practical implementation of prevention activities in schools. Following discussions focused on reflections regarding the observed intervention and potential implications for the design of the nicotine prevention program.

**Supplementary Note S2:**

**Integrating divergent answers or preferences between groups of interview participants**

The development process drew on interviews with two participant groups, students and stakeholders. Divergent responses were explicitly welcomed and carefully considered. In most instances, differences between groups were not contradictory, but instead reflected their respective perspectives and roles. For example, some children described smoking in the family environment as normal and did not necessarily perceive practices such as indoor smoking as problematic, whereas teachers identified such normalization as a substantial barrier to smoking prevention.

A similar pattern emerged in the review of intervention materials. Children occasionally indicated that certain parts of the materials were difficult to understand, while pedagogical stakeholders provided valuable input on children’s prior knowledge and basis for understanding.

However, when intervention content had to be reduced because of time constraints, some topics valued by stakeholders had to be removed. Under these circumstances, we prioritized content primarily on the basis of children’s observed engagement with the materials.

**Supplementary Table S1:**

*Needs assessment interviews and survey study: Illustrative quotes*

| **Theme** | **Sub-category** | **Source** | **German (verbatim)** | **English Translation** |
| --- | --- | --- | --- | --- |
| A1: Economic deprivation / social problems | Financial hardship / Family smoking | girl,  low SES school | Einmal hat mein Vater so eine Zehnerpackung von Zigaretten gekauft, aber an der Tankstelle, das hat, glaube ich, so 125 Euro dann am Ende gekostet und er hat dann uns noch nichtmal ein kleines Eis gekauft. | *Once my father bought a ten-pack of cigarettes at the petrol station; I think it ended up costing about 125 euros and he didn't even buy us a small ice cream [due to financial constraints].* |
|  | Financial hardship | boy, low SES school | die konnten früher hatten die nicht so viel Geld und so, um in die Moschee zu gehen, deswegen die konnten sich das nicht leisten, aber die wollen das ich  wenigsten das schaffe und alles so mache. | *Back then they didn't have much money to go to the mosque [to pray]; they couldn't afford it; but they want me to do it at least.* |
| B1: Exposure to smoking/vaping | Family | girl,  low SES school | Also meine Eltern rauchen ständig die, mein Vater hat früher die geraucht, dann hat er wieder zu denen gewechselt und meine große Schwester, die glaube ich gerade erst 14 ist, raucht auch schon die. | *My parents smoke constantly; my big sister, who I think just turned 14, already smokes too.* |
|  | Family | boy, low SES school | Meine Eltern rauchen Zigaretten, mein Bruder raucht eigentlich so, also hat auch ziemlich eigentlich alles | *My parents smoke cigarettes, my brother smokes pretty much everything.* |
|  | Family | boy, very low SES school | Also meine Mutter, mein Vater, mein Onkel, also fast, viele aus meiner Familie. Meine Mutter raucht Vape und Zigarette, ja. | *My mother, my father, my uncle — basically almost -, lots of my family. My mother smokes vape and cigarettes, yes.* |
|  | Home | boy, low SES school | Wir haben halt keinen Balkon, weil wir sind im Erdgeschoss und unsere Wohnung an sich hat auch keinen Balkon aber eigentlich, also mein Vater raucht immer im Wohnzimmer | *We don't have a balcony because we're on the ground floor and our flat doesn't actually have a balcony, so my father always smokes in the living room.* |
|  | Home | girl,  low SES school | Wo fast überall eine Ziga/, obwohl immer in der Küche ungefähr drei Packungen Zigaretten liegen und auf jeden Fall ungefähr so zwei Feuerzeuge | *Where there's almost always a ciga-/, even though there are always about three packs of cigarettes in the kitchen and definitely about two lighters.* |
|  | Public spaces | boy, low SES school | Ja natürlich. Sieht man hier überall | *[Seen people vaping/smoking]. Yes of course. You see it everywhere here.* |
|  | Public spaces | girl, very low SES school | E-Zigarette, ja, sehr viele an der Bushaltestelle, immer wenn ich (unv.) Schule zurückkehre nach Hause, sehe ich sehr viele und ja und meistens sehe ich halt diese Zigaretten. | *E-cigarettes, yes, very many at the bus stop; whenever I go home from school I see very many.* |
|  | Public spaces | girl, very low SES school | Auf jeden Fall draußen kann ich sehr oft beobachten. (...) Parks, generell vor Läden, genau (...) sehr oft. | *I can definitely observe it outside very often. Parks, in front of shops generally — very often.* |
|  | School (students) | boy, low SES school | Der eine, also er hatte immer die Vape hier und wenn die Lehrer weggekuckt haben, hat er so gezogen | *This one guy always had his vape right here, and when the teachers looked away he'd take a drag.* |
|  | School (students) | boy, very low SES school | Boah, es gibt schon, glaube ich, dreizehnjährige, ja. (...) Heimlich, glaube ich, auf den Toiletten. | *Whoa, there are already thirteen-year-olds I think [who smoke/vape at schoo]. Secretly, I think, in the toilets.* |
|  | School (students) | girl, very low SES school | Ältere schon hier rauchen (...) weil ich die vor der Schule halt sehe nach Schulschluss mit Schultasche dann halt zum Beispiel das in der Hand, am meisten halt das hier, die Vape/ | *Older ones [students] do smoke here because I see them in front of the school after school with their schoolbag and the vape in hand.* |
|  | School (teachers) | boy, low SES school | (Lehrername) [...] vor dem (Ort) hat geraucht mit (Lehrername). | *(Teacher name) smoked in front of (place) with (teacher name).* |
| B2: Online/social media exposure | Advertising | boy, low SES school | Nein, ich habe mal Werbung zu E-Zigaretten gesehen, aber zu normalen Zigaretten nicht. / Ne, das ist irgendwie so eine Werbung, meistens verschieden, wo einfach so eine Person rumläuft und dann so, der das benutzt und dann irgendwie so Musik kommt oder sowas und sich dann super gut fühlt | *I've seen adverts for e-cigarettes, but not for normal cigarettes. / It's like an advert where a person just walks around using it and then some music plays and they feel really great.* |
|  | Social media/ Influencers | girl, very low SES school | So die machen solche Videos halt zum Beispiel von (unv.)/ ich sage jetzt einfach von dieser E-Zigaretten zum Beispiel was für ein Geschmack die haben und danach die zeigen es so halt, dass man es so kaufen will so. | *They make those videos about e-cigarettes, for example what flavours they have, and then they show it in a way that makes you want to buy it.* |
|  | Social media/ Advertising | girl, very low SES school | Youtube. / Ich spiele auch solche Spiele, dann kommt ja auch immer Werbung und dann hab/ aber ich habe die glaube ich nur zweimal gesehen oder so gesehen, diese. | *YouTube. / I also play those games, and adverts always come up, but I think I've only seen those ones twice or so.* |
| B3: Barriers to being smoke-free | Wanting to be cool / belonging | boy, low SES school | Also ich glaube die meisten Kinder fangen ja an zu rauchen, weil sie entweder gestresst sind oder ein Problem mit etwas haben oder sich einfach einer Truppe anschließen wollen | *I think most kids start smoking because they're either stressed, have a problem, or simply want to join a group.* |
|  | Wanting to be cool / belonging | girl,  low SES school | Ist halt das Problem, es könnte sein, dass irgendwelche anfangen zu rauchen und dann, wie ich schon gesagt habe, dass man sich dann ausgeschlossen also fühlt | *That's the problem: some might start smoking and then, as I said, you end up feeling excluded.* |
|  | Wanting to be cool / belonging | boy, very low SES school | Weil die sich cool fühlen wollen und (...) weil die vielleicht ein bisschen, na das ist halt so komisch, weil (...) ich weiß nicht, wie ich es erklären soll (...) | *Because they want to feel cool and (...) I don't know how to explain it.* |
|  | Peer pressure | boy, very low SES school | Außer, sagen wir mal jetzt (...) so ein Freund, der mir wichtig im Leben ist, würde mir so sagen 'Rauch mal, sonst Freundschaft beendet' dann würde ich halt mal probieren | *Unless a friend who is important to me said 'Smoke or the friendship is over,' then I'd probably try it.* |
|  | Peer pressure | boy, low SES school | was mich anspornen würde, wenn ich größer bin, zum Rauchen, wäre das wenn ALLE im Umfeld rauchen würden | *What would push me to smoke when I'm older is if EVERYONE around me smoked.* |
|  | Stress | girl,  low SES school | Wenn ich richtig Stress habe, mir richtig kacke geht. Wenn ich irgendwas Böses machen will und wenn meine Freunde dann sagen 'Komm rauch mal eine Zigarette', dann würde ich es, glaube ich, machen | *If I'm really stressed, if things are really shit. If my friends say 'Come on, smoke a cigarette,' then I think I'd do it.* |
|  | Stress | girl,  low SES school | Also entweder, ich glaube, Kinder vor allem wegen ihrer Freundesgruppe oder wegen anderen Menschen, Depression kann auch, glaube ich, manchmal ein Grund sein oder Stress generell | *Children mainly because of their friend group or other people; depression can also be a reason I think, or stress in general.* |
|  | Stress | girl, very low SES school | Ich sag mal so, wenn man raucht gibt es ja auch manchmal Gründe, wegen halt zum Beispiel, was man in Vergangenheit hatte oder so oder Schmerzen, dein Herz (oder?) so, dass man die wegraucht, also mit den Stoff einfach so, wie sagt man das, so verschwinden will, also das das weggeht (...) | *When you smoke there are sometimes reasons, like things from the past or pain, your heart, so you smoke it away — you want it to just disappear.* |
| B4: Enablers of being smoke-free | Health / fitness | boy, low SES school | ich bin ausgebucht in einer Woche, ich habe meine Aktivitäten, Fußball, Volleyball, Gitarre und ich glaube, das würde mir auch gar nichts bringen zu rauchen und ich will ja auch sportlich bleiben und ich will mein, sagen wir mal, Leben nicht gefährden | *I'm booked up all week — football, volleyball, guitar — I think smoking wouldn't do anything for me; I want to stay sporty and not endanger my life.* |
|  | Health / costs | boy, very low SES school | Wenn man es sein lässt, hat man eine bessere Lunge, erstens, zweitens man hat mehr Geld, man spart es und ja man bleibt mehr gesund, als wenn man raucht. | *If you refrain from it: first, you have better lungs; second, you have more money; and you stay healthier.* |
|  | Health / costs | girl,  low SES school | Ich würde rauchfrei bleiben, weil erstens dann könnte ich mir eine Menge Geld sparen und zweitens dann bin ich auch viel gesünder | *I would stay smoke-free because, first, I could save a lot of money, and second, I'd be much healthier.* |
|  | Smell / addiction fear | girl,  low SES school | Also ich mag den Geruch nicht, ich habe Angst beim ersten Mal mich irgendwie zu verschlucken oder irgendwie, dass es nicht gut läuft, ich habe einfach Angst davor, dass ich süchtig danach werde. | *I don't like the smell; I'm afraid of choking the first time; I'm just afraid of becoming addicted.* |
|  | Friends | girl,  low SES school | ich gehe bald zu Leichtathletik, aber also ich hoffe, da wird niemand sein, aber ich glaube nicht, weil ich gehe mit meiner besten Freundin hin und die ist auch gegen Rauchen und ich glaube, wir würden uns dann so gegenseitig unterstützen. | *I'm starting athletics soon, and I hope no one there smokes, because I'm going with my best friend who's also against smoking, and I think we'd support each other.* |
|  | Family expectations | girl, very low SES school | Also meine/ ich hab es halt, mein Vater und meine Mutter hat mir das mal gesagt, du sollst am liebsten gar nicht rauchen, auch wenn du älter bist, auch wenn du dreißig oder so bist, einfach gar nicht rauchen. | *My father and mother told me: you should preferably never smoke, even when you're older, even when you're thirty — just never smoke.* |
|  | Family expectations | girl, very low SES school | Zum Beispiel die Unterstützung von meiner Familie, dass sie es sagen, dass ich es nicht mehr machen soll oder dass ich so daran denke, wie schlimm es eigentlich für mich ist. Dann/ dann könnte ich es schon lassen so. | *For example the support of my family, telling me I shouldn't do it, or me thinking about how bad it actually is. Then I could manage to leave it.* |
| B5: Cigarettes less attractive (odor, cost, health) | Odor | boy, very low SES school | Und allein der Geruch, der nervt mich auch immer. / Das man immer wieder Geld ausgibt dafür so ja und dabei noch, das ist, da gibt es ja nichts Positives dabei, wenn man raucht, nur Negatives | *The smell alone always annoys me. You keep spending money on it — there's nothing positive about smoking, only negative.* |
|  | Odor, cost | boy, low SES school | Zigaretten sind, finde ich, eklig, weil die so stinken | *I think cigarettes are disgusting because they stink so much.* |
|  | Odor, health | girl,  low SES school | Das ist ungesund und ja, und es stinkt. | *It's unhealthy, and yes, it stinks.* |
|  | Odor | girl, very low SES school | Bei den/ halt Rosanen ist der Geruch ist schön, (...). | *With the pink ones [Vapes as opposed to cigarettes] the smell is nice, (..).* |
| B6: Vapes highly attractive (flavors, appearance, cost, access) | Odor / flavors | boy, low SES school | mein Bruder hat so andere Geschmäcker und wenn er davon zieht pustet er manchmal in mein Gesicht und das riecht, da muss ich auch ehrlich sein, sehr gut, also sehr geil | *My brother has different flavours and when he takes a drag he sometimes blows it in my face, and it smells — I have to be honest — really good.* |
|  | Flavors | girl, very low SES school | Zum Beispiel sehe, das riecht dann immer so nach Himbeere so richtig lecker, dann denke ich mir, oh das werde ich auch mal machen, dann denke ich mir aber mein Gehirn nein, das werde ich nicht machen | *It always smells like raspberry, really tasty; then I think oh I'll do that too, but then my brain says no.* |
|  | Appearance / access | girl, very low SES school | Weil die sehr/ ich bin ehrlich an der Tankstelle sind, wenn man reinkommt, ja wo eine Kasse ist und (unv.) diese Dinger hier, diese Vape und es gibt dann manchmal diese, nicht diese Papier wo/ da steht dann Vape und so und dann sind immer hier drin diese Farben und so und man kann einfach nehmen und so. | *At the petrol station when you go in, where the till is — these vapes, the colors are always in there and you can just take them.* |
|  | Access | boy, low SES school | Ich würde ganz leicht herkriegen. Ich habe Freunde, die mir das bestimmt, also ich kenne einen Laden, der nicht meine Eltern kennt, aber der immer an kleine Kinder Sachen verkauft | *I could get hold of them really easily. I know a shop that doesn't know my parents but always sells things to little kids.* |
|  | Flavors | girl,  low SES school | Also bei einer E-Zigarette wäre ich mir, glaub ich, da so bei fünf, bei einer E-Zigarette wäre ich mir da bei acht und bei einer Shisha wäre ich mir bei zehn. / Weil bei einer E-Zigarette, da gibt es ja auch verschiedene Geschmackrichtungen deswegen bin ich mir da auch nicht mehr so sicher. | *For an e-cigarette I'd say about five [rating on a scale: confidence of refusing; 1-10]. Because with an e-cigarette there are different flavours, so I'm not so sure anymore.* |
| B7: Strong cigarette refusal but vapes tempting | Flavors | girl, very low SES school | Weil wenn ich ehrlich bin, ich mag eigentlich gar keins, aber die mag ich am meisten die Vape, Vapes, Vapes und deswegen bin ich da bei einer neun, halt weil ich da näher rankommen würde sehr wenig, als bei einer Zigarette oder Shisha oder. | *To be honest, I don't actually like any of them, but I like vapes the most — that's why I'm at a nine [confidence of refusing].* |
|  | Taste | girl,  low SES school | Ich weiß nicht genau, wie ungesund/ also ich glaube, dass E-Zigaretten von Hören jetzt, glaube ich ein bisschen gesünder sind, sage ich mal, als Shisha oder Zigaretten / Ich glaube schon, ja. | *I think from what I've heard that e-cigarettes are maybe a bit healthier than shisha or cigarettes. I think so, yes. [would say yes more easily for e-cigarettes than for cigarettes]* |
| B8: Uncertainty about harm of vaping | Health | boy, low SES school | Sind die E-Zigaretten gleich schlimm wie die normalen Zigaretten. | *Are e-cigarettes just as bad as normal cigarettes?* |
|  | Health | girl,  low SES school | Also schlimmer als die anderen. Weil es ist tödlich und die anderen keine Ahnung | *Worse than the others [cigarettes]. Because it's deadly and the others [e-cigarettes] — no idea. [cigarettes deadly; others unknown]* |
| C1: Gender-specific motives to smoke/vape | Girls — stress / coping; Boys — status | boy, low SES school | Ja, Mädchen eher wegen Stress und Jungs mehr wegen falschen Freunden | *Yes, girls [vape/smoke] more because of stress and boys more because of wrong friends.* |
|  | Girls — stress / coping; Boys - taste | boy, low SES school | Also bei den Jungs, glaube ich, den Geschmack, bei den Mädchen auch Geschmack, aber auch wegen dem Stress, weil zum Beispiel, sagen wir mal du bist verheiratet und du musst putzen die ganze Zeit und du bist so gestresst | *[Motives for vaping/smoking]. For the boys the taste; for the girls also the taste, but also because of stress — say you're married and you have to clean all the time and you're so stressed.* |
|  | Boys — impressing girls | girl, very low SES school | Die wollen auf cool tun. / Weil die auf cool tun wollen. | *[Motives for vaping/smoking]. They [boys] want to act cool. Because they want to act cool.* |
|  | Boys — coolness / status | girl, very low SES school | Ich glaube, sie wollen die Mädchen einfach beeindrucken und so, ja. Genau, das ist meine Vermutung. | *[Motives for vaping/smoking]. I think They [boys] they just want to impress the girls.* |
|  | Boys — coolnesss / status | boy, low SES school | Also ich glaube die meisten wollen sich damit cool fühlen | *I think most of them just want to feel cool with it.* |
|  | Boys — fitness / sports | boy, low SES school | ich bin ausgebucht in einer Woche, ich habe meine Aktivitäten, Fußball, Volleyball, Gitarre und ich glaube, das würde mir auch gar nichts bringen zu rauchen und ich will ja auch sportlich bleiben | *I'm booked up all week — football, volleyball, guitar — I want to stay sporty.* |
|  | Boys — fitness / sports | boy, very low SES school | Ja, das man halt nicht mehr stärker Ausdauer mit der Lunge hat, also das man halt nicht so lange rennen kann eigentlich nur, weil die Lunge dann irgendwann schlapp macht. | *You don't have as much endurance with your lungs — you can't run as long because the lungs eventually give out.* |
| C3: SES: SES-specific: sports club membership | Very low SES school — no clubs | boy, very low SES school | Ne, ich hab keinen Verein, wo ich hingehe. Ich bin eigentlich meiste Zeit nur zu Hause und in der Schule halt. | *No, I don't have a club I go to. I'm just at home and at school most of the time.* |
|  | Less burdened (low SES) school - club memberships | boy, low SES school | Ich habe dreimal in der Woche Fußball, dann Volleyball am Freitag, also Montag, Dienstag und Donnerstag habe ich Fußball, Mittwoch habe ich Gitarre / Also viele/ alle Jungs aus meiner Klasse sind in einem Fußballverein | *I have football three times a week, then volleyball on Friday. Most/all the boys in my class are in a football club.* |
| C4: Religious beliefs / culture | Islam | boy, low SES school | außerdem würde er nicht wegen seiner Reli/, also wegen unserer Religion, wir dürfen auch in unserer Religion eigentlich nicht rauchen, ja (..). Ich bin halt Muslime, also ich bin ein Moslem und wir dürfen halt nicht rauchen zum Beispiel oder wir dürfen nichts essen, was nicht richtig geschlachtet wurde wie bei uns ja | *Because of our religion — we're actually not allowed to smoke in our religion. I'm Muslim and we're not allowed to smoke, for example, or eat anything that hasn't been properly slaughtered.* |
|  | Islam / faith | boy, low SES school | Hundertprozentig / Ich würde es nicht sagen, aber es wäre einer der Gründe, warum ich nicht rauchen würde / Ja. (...) Weil ich bin ein sehr gläubiger Mensch | *One hundred percent [would never smoke/vape]. It would be one of the reasons why I wouldn't smoke. Because I'm a very devout person.* |
| D1: Feasibility of intervention /ease of integration into regular teaching and routine school practice | Teacher | female, low SES school | Gute und praxisnahe Ergänzung; Stimmen von außen haben eine andere Wirkung | *Good and practical addition; voices from outside have a different effect* |
|  | Teacher | female, very low SES school | Kurzweilig und interessant | *Entertaining and interesting* |
| D3: Possible improvements of program | Teacher | female, low SES school | Auf einfache Sprache achten; Möglicherweise mehrsprachigen Fragebogen oder Übersetzungshilfe | *Pay attention to simple language; Possibly a multilingual questionnaire or translation aid* |
|  | Students | girl,  low SES school | Alles war gut :) | *Everything was great :)* |
|  | Students | boy, very low SES school | Es ist schon perfekt. | *It is already perfect.* |

Cells A1–C4 present the transcribed student needs assessment, and cells D1–D3 present open-ended responses to items from the post-intervention survey used in the feasibility study.
