# Supplementary material for: Development of a school program for vaping and smoking prevention and protocol for a cluster randomized controlled trial in fifth grade students
Source: Sci Rep. 2026 May 7;16:13263. doi: 10.1038/s41598-026-45720-w (PMC13153207; doi:10.1038/s41598-026-45720-w)
Supplement: Supplementary file 2 — Supplementary Information 2. [file 41598_2026_45720_MOESM2_ESM.docx]

**Supplementary File 2: Study Protocol for Cluster-Randomized Control Trial**

**Supplementary Table S2:***Summary of measurements used in student questionnaires with references*

| **Measurement** | **T0** | **T1** ^a^ | **T2** | **T3** | **T4** | **Tn** |
| --- | --- | --- | --- | --- | --- | --- |
| **Socio-demographics / Covariates** |  |  |  |  |  |  |
| Age, gender | X | X | X | X | X | X |
| Social determinants of health [1]: Country of origin, objective SES [2, 3], subjective SES [4, 5], ethnicity/racial identity [1], language [6], religion [7], general health [8] | X |  |  |  |  |  |
| Vaping/smoking of relevant others [9], parental disapproval of vaping/smoking [10], accessibility^b^, school attachment [11], self-esteem [12], satisfaction with looks [13], distress^b^ [14] | X |  |  |  |  |  |
| Careful responding [15] | X | X | X | XX | X |  |
| **Outcomes** |  |  |  |  |  |  |
| Intention to be vape- and smoke-free [16, 17], susceptibility to vaping and smoking [18–20] | X | X | X | X | X | X |
| Lifetime prevalence, 12-months-prevalence, current smoking, regular smoking/vaping, 30-day-frequency [21, 22] | X |  | X | X | X | X |
| **Proposed mechanisms (mediators)** |  |  |  |  |  |  |
| Personal agency: self-efficacy to be vape- and smoke-free [16, 17], refusal self-efficacy [23], refusal self-concept [24, 25] ^b^ | X | X | X | X | X | X ^a^ |
| Action planning [26] | X | X | X | X | X | X ^a^ |
| Descriptive and injunctive norms towards being vape- and smoke-free [16, 17] | X | X | X | X | X | X ^a^ |
| Attitude towards being vape- and smoke-free (direct measure) [16, 17] | X | X | X | X | X | X ^a^ |
| Outcome expectations (indirect attitude measure) [10, 16, 17, 27–29] | X | X | X | X | X |  |
| Stress and problem coping self-efficacy [16], stress and problem coping planning [26], affect (LE-PANAVA) [30]^a^, anxiety [31]^a^ |  |  |  | XX | X | X^a^ |
| **Program evaluation** |  |  |  |  |  |  |
| Satisfaction [32]: Liking of the program; program should be offered to other students |  | X | X | X |  |  |
| Careful responding /attention [15] | X | X | X | X | X | X |

T0=Baseline: conducted approximately one week or immediately before the first core workshop, T1: immediately after core workshops 1 and 2, T2: 3 months post-intervention, T3: 6 months post-intervention, T4: 12 months post-intervention, Tn= optional follow-up surveys up to the age of 21. “XX” indicates assessments conducted both immediately before and after the workshop in the treatment condition (T3).
^a^ = measured only of the intervention arm participants. ^b^ = Questionnaire measure developed during the feasibility study for use in this study based named sources.

**Supplementary Table S3:**
*Overview of T0-T4 measurements and question order at each assessment timepoint*

| Timepoint | Student survey | Teacher survey | Interventionist survey^a^ |
| --- | --- | --- | --- |
| T0 Before implementation of the teaching unit | A: Intro  B: Vaping/ smoking behavior and intention & attention check  C: Determinants of vaping/smoking behavior  G: Social determinants of health  H: Question on careful responding |  |  |
| T1 After teaching unit | A: Intro  B: Vaping/ smoking behavior and intention & attention check  C: Determinants of vaping/smoking behavior (abridged)  F: Evaluation^a^  H: Question on careful responding | A: Intro  B: Organizational information  C: Evaluation^a^  D: General report^a^  E: Sociodemographics  F: Vaping/Smoking behavior  G: Social determinants of health | A: Organizational information  B: Adherence (T1)  C: Competence (T1)  D: General report |
| T2 After teaching unit^a^ |  | A: Intro  H: Organizational information/Adherence booster  I: Organizational information/Competence booster  C: Evaluation  D: General report E: Sociodemographics  F: Vaping/Smoking behavior  G: Social determinants of health | A: Organizational information  E: Adherence (T2)  F: Competence (T2)  D: General report |
| T3 Before teaching unit | A: Intro  B: Vaping/ smoking behavior and intention & attention check  C: Determinants of vaping/smoking behavior (abridged)  D^a^: Affect and anxiety  E: Coping with stress and problems  H: Question on careful responding |  |  |
| T3 After teaching unit^a^ | A: Intro  D: Coping with stress and problems  E: Affect and anxiety  F: Evaluation  H: Question on careful responding |  | A: Organizational information  G: Adherence (T3)  H: Competence (T3)  D: General report |
| T4: 12-month follow-up | A: Intro  B: Vaping/ smoking behavior and intention & attention check  C: Determinants of vaping/smoking behavior (abridged) D: Coping with stress and problems  H: Question on careful responding |  |  |

T0=Baseline: conducted approximately one week or immediately before the first core workshop, T1: immediately after core workshops 1 and 2, T2: 3 months post-intervention, T3: 6 months post-intervention, T4: 12 months post-intervention.

^a^ = measured only of the intervention arm participants.

**Main questionnaire for students**

At baseline, students will be instructed to complete a work sheet with sample items to familiarize themselves with the question format and survey-specific terminology. In particular, they view a mind-map demonstrating the use of the term “smoking” or “being smoke-free” as umbrella terms for use of/abstinence from any tobacco or vaping product.

General note:

If not otherwise stated, response options range from

□ strongly disagree □ somewhat disagree □ partially agree, partially disagree □ somewhat agree □ fully agree

Measures for social determinants of health are presented in a separate manuscript.

**Supplementary Table S4:**

*Student questionnaires: measured constructs, number of items and example items translated in english*

| Part | Variable | Number of Items | Item / Validated scale | Sources |
| --- | --- | --- | --- | --- |
| A: Intro | Pseudonym | 1 | **Before we can start, we need your participant code Please enter your code, which you created with the help sheet, here**: _______________ |  |
|  | Age |  | **The used measures will be published as part of a separate manuscript.** |  |
|  | Gender |  | **The used measures will be published as part of a separate manuscript.** |  |
| B: Vaping/ smoking behavior and intention & attention check | Vaping/Smoking behavior Lifetime prevalence | 1 | **Which of the following products have you ever smoked? (multiple choice possible)**  □ Cigarettes □ (Disposable) vapes / vape (e.g.: Elf Bar 600) □ E-cigarettes (reusable / refillable, e.g. with tank) □ Water pipe / shisha □ Tobacco heater (e.g. IQOS, Glo) □ Cigars, cigarillos □ Pipe □ None of the above | **Adaptation of KiGGs wave 1 (inclusion of further product categories)** [21, 33] |
|  | Vaping/Smoking behavior 12-month prevalence | 1 | **Which of the following products have you smoked in the last 12 months? (multiple choice possible)**  □ Cigarettes □ (Disposable) vapes / vape (e.g.: Elf Bar 600) □ E-cigarettes (reusable / refillable, e.g. with tank) □ Water pipe / shisha □ Tobacco heater (e.g. IQOS, Glo) □ Cigars, cigarillos □ Pipe □ None of the above |  |
|  | Current/Regular Vaping/Smoking | 1 | **How often do you currently smoke the products mentioned?**  □ Cigarettes □ (Disposable) vapes / vape (e.g.: Elf Bar 600) □ E-cigarettes (reusable / refillable, e.g. with tank) □ Water pipe / shisha □ Tobacco heater (e.g. IQOS, Glo) □ Cigars, cigarillos □ Pipe  *Scale for each product*  Not at all □ Less than once a week □ Once a week □ Several times a week □ Daily |  |
|  | Vaping/Smoking behavior 30-day consumption frequency | 1 | **How many days have you smoked the above products in the last month?**  □ Cigarettes □ (Disposable) vapes / vape (e.g.: Elf Bar 600) □ E-cigarettes (reusable / refillable, e.g. with tank) □ Water pipe / shisha □ Tobacco heater (e.g. IQOS, Glo) □ Cigars, cigarillos □ Pipe  *Input option for each product* |  |
|  | Intention to be vape-/smoke-free | 2 | **How much do you agree?**  **I want to be smoke-free (that is, not smoke) for the next six months.**  □ Do not agree at all □ Rather disagree □ Partly agree, partly disagree □ Rather agree □ Fully agree | [16, 17] |
|  | Susceptibility to vaping/smoking | 3 | **Do you think you will try smoking in the future?**  □ Definitely not □ Probably not □ Probably yes □ Definitely | Adapted from [18–20]: Choi et. al, 2001: display logic, Strong et. al, 2015: coding |
|  | Attention check | 1 | **It is very important to us that you read our questions carefully.**  **Please mark response option "Definitely" here.**  **I am currently answering a questionnaire.**  □ Definitely not □ Probably not □ Probably yes □ Definitely |  |
| C: Determinants of vaping/smoking behavior | Self-efficacy | 2 | **How much do you agree?**  **▪ I can be smoke-free in the next six months.** | [16, 17] |
|  | Ability self-concept: saying "no | 3 | **The following questions relate to your ability to refuse when someone offers you something to smoke. Please rate yourself:**  **▪ It's easy for me to assert myself when someone tries to persuade me to smoke.** | Adapted from: [24, 25] |
|  | Refusal self-efficacy | 2 | **Someone offers you to smoke in the next 6 months.**  **▪ I have the confidence to turn down an offer to smoke, even if my friends laugh at me for it** | [23] |
|  | Action planning | 2 | **How much do you agree?**  **I have a plan for what I say when someone makes me a smoking offer.** | [26] |
|  | Normative beliefs | 2 | **How many of the people you care about smoke?**  □ none □ only a few □ half □ most, but not all □ all | [16, 17] |
|  | Vaping/smoking of close others: parents/best friends | 4 | **Do any of the following people smoke?**  □ First parent (e.g. mother) | [9] |
|  | Attitude (direct measure) | 2 | **Being smoke-free next year would be...**  **▪ Very good** | [16, 17] |
|  | Outcome expectations: Being vape- and smoke-free (indirect attitude measure) | 9 | **How much do you agree with the following statements?**  **If I am smoke-free for the next six months ...**  **... my friends don't want me around anymore.** | [16, 17, 27] |
|  | Outcome expectations: Smoking/vaping (indirect attitude measure) | 7 | **How much do you agree with the following statements?**  **If I smoke next year,**  **... it will help me to relax.** | [16, 17, 34] |
|  | Perceived availability of smoking products^b^ | 1 | **Do you know a store that would sell you smoking products?**  □ yes □ no □ don't know |  |
|  | School attachment^b^ | 3 | **How much do you agree?**  **▪ I like my school.** | [11] |
|  | Self-esteem^b^ | 1 | **How much do you agree?**  **▪ I have a high level of self-esteem.** | [12] |
|  | Satisfaction with your own appearance^b^ | 1 | ▪ **I look good.** | [13] |
|  | Distress^b^ | 2 | **How often have you had the following complaints / feelings in the last 2 weeks?**  **▪ Stress**  □ Not at all □ On individual days □ On more than half of the days □ Almost every day | Developed and used for the first time during the feasibility study;  Response anchor adapted from  Patient Health Questionnaire PHQ-4: [14] |
| D: Anxiety and affect^a^ | Anxiety | 4 | **How do you feel at the moment?**  **▪ I am tense.** | Adapted from [31] |
|  | Affect | 5 | **How do you feel at the moment?**  *Each sub-item with Likert-scale from 1-7 where 1 is the strongest rating for the first term and 7 is the strongest rating for the second term*  *Each term is accompanied by an emoticon.*  ▪ **calm – nervous** | [30] |
| E: Coping with stress and problems | Stress and problem coping self-efficacy | 2 | **How much do you agree?**  **▪ I can deal well with difficult situations.** | Adapted from [16, 17] |
|  | Stress and problem coping planning | 2 | **How much do you agree?**  **▪ I have a plan for what I will do if I have a difficult problem.** | Adapted from [26] |
| F: Evaluation^a^ | Satisfaction with intervention | 2 | *How much did you enjoy the program?*  □ I didn't like it at all □ I rather didn't like it □ I partly liked it □ I rather liked it □ I liked it a lot | Adapted from [32] |
| G: Social determinants of health | Social determinants of health |  | **The used measures will be published as part of a separate manuscript.** |  |
| H: Question on careful responding | Honesty / Careful responding | 1 | It is important to us that we only use serious and honest answers to this questionnaire. Be completely honest: did you answer the questions seriously?  □ yes □ no | [15] |

T0=Baseline: conducted approximately one week or immediately before the first core workshop, T1: immediately after core workshops 1 and 2, T2: 3 months post-intervention, T3: 6 months post-intervention, T4: 12 months post-intervention.

^a^ = measured only of the intervention arm participants.
^b^ = measured at base-line only.

**Main questionnaire for teachers**

General note:

If not otherwise stated, response options range from

□ strongly disagree □ somewhat disagree □ partially agree, partially disagree □ somewhat agree □ fully agree

Measures for social determinants of health are presented in a separate manuscript.

**Supplementary Table S5:**
*Teacher questionnaires: measured constructs, number of items and example items translated in English*

| Part | Variable | Number of Items | Item / Validated scale | Sources |
| --- | --- | --- | --- | --- |
| A: Intro | Pseudonym | 1 | **Please first enter the code that you created with the help sheet.** _______________ |  |
| B: Organizational information | Class size | 1 | **How many children attend your class?**  Dropdown: 0-30 |  |
|  | Number of children present | 1 | **How many children were present in class today?**  Dropdown: 0-30 |  |
|  | Intro | 1 | The following questions relate to the teaching program that you have just accompanied. |  |
|  | Unit | 1 | **Which unit was taught today?**  □ Core workshop part 1 (90 min) □ Core workshop part 2 (90 min) □ Core workshop part 1 and 2 (180 min) |  |
|  | Grading | 2 | **Did you use the collaboration in the project for a performance review/grading?**  □ Yes □ No  **Please report briefly on what you** have **evaluated.** ________  *Only appears if the previous question "grading" was answered with "Yes"* |  |
| C: Evaluation | Satisfaction with intervention | 2 | **How much did you like the program?** □ I didn't like it at all □ I rather didn't like it □ I liked it partly □ I rather liked it □ I liked it a lot | [32] |
| D: General report | Positive and negative events | 2 | **What positive events occurred while teaching the unit?**  *_________________________________________* |  |
| E: Sociodemographics | Occupation at the school | 1 | **I work at the school as:**  □ Teacher □ Educator □ School social worker □ School psychologist □ Integration assistant □ Head of school □ Secretary □ Another function, namely:_____________ |  |
|  | Age | 1 | **How old are you? _______________________** |  |
|  | Gender | 1 | **Which gender do you feel you belong to?**  □ Female □ Male □ Prefer self-identification as: _____________ □ Not specified |  |
| F: Vaping/Smoking behavior | Vaping/Smoking behavior 12-month prevalence | 1 | **Which of the following products have you smoked in the last 12 months? (multiple choice possible)**  □ Cigarettes □ (Disposable) vapes / vape (e.g.: Elf Bar 600) □ E-cigarettes (reusable / refillable, e.g. with tank) □ Water pipe / shisha □ Tobacco heater (e.g. IQOS, Glo) □ Cigars, cigarillos □ Pipe □ None of the above | **Adaptation of KiGGs wave 1 (inclusion of further product categories)** [21, 33] |
|  | Current/Regular Vaping/Smoking | 1 | **How often do you currently smoke the products mentioned?**  □ Cigarettes □ (Disposable) vapes / vape (e.g.: Elf Bar 600) □ E-cigarettes (reusable / refillable, e.g. with tank) □ Water pipe / shisha □ Tobacco heater (e.g. IQOS, Glo) □ Cigars, cigarillos □ Pipe  *Scale for each product*  Not at all □ Less than once a week □ Once a week □ Several times a week □ Daily |  |
| G: Social determinants of health | Social determinants of health |  | **The used measures will be published as part of a separate manuscript.** |  |
| H: Organizational information/Adherence booster | Booster unit | 1 | **Have you taught the booster unit?**  □ yes  □ no |  |
|  | Time of filling in survey | 1 | **When are you filling out this questionnaire?**  □ right after having taught the booster teaching unit  □ a few days after having taught the booster unit  □ while the study team is teaching the booster unit  □ after the study team taught the booster unit |  |
|  | Intro | 1 | **The following questions relate to the teaching program you implemented.** |  |
|  | Number of days | 1 | **The crafting in particular is time-consuming and can take longer than a school lesson. Did you carry out the program on one or more days?**  □ one day  □ two days  □ three days |  |
|  | Date(s) | 3 | **Please indicate here when you completed the program:**  **___________________________________** |  |
|  |  |  | **Please enter the second date on which you completed the program:** *(Displayed only if two/three days chosen)*  **___________________________** |  |
|  |  |  | **Please enter the third date on which you completed the program:** *(Displayed only if two/three days chosen)*  **_______________________________** |  |
|  | Subject | 1 | **In which lessons was the program integrated? (e.g. science)**  **__________________________** |  |
|  | Class size | 1 | **How many children attend the class?**  Dropdown: 0-30 |  |
|  | Number of children present | 1 | **How many children were present in class today?**  Dropdown: 0-30 |  |
|  | Duration booster | 1 | **How long did you teach the program?**  □ 30-45 min  □ 45-60 min  □ 60-75 min  □ 75-90 min  □ more than 90 min |  |
|  | Materials used in booster | 1 | **What materials did you use?**  □ Powerpoint presentation  □ Preparation guide / script for content  □ Photos sent |  |
|  | Dose block-wise | 1 | **What percentage of the individual content was conveyed?**   - Block 1: Review of the content from Workshop 1 - Block 2: Personal experiences with smoking offers - Block 3: Ways to say “no” - Block 4: Group work: Advertising posters   *The following answer options for each block:*  □ 0-20% □ 20-40% □ 40-60% □ 60-80% □ 80-100% □ Not planned today |  |
| I: Organizational information/ Competence booster | Quality | 1 | **The workshop went well** | [35] |
|  | Quality block-wise | 1 | **How satisfied were you with the flow of the individual content?**     - *Block 1: Review of the content from Workshop 1* - *Block 2: Personal experiences with smoking offers* - *Block 3: Ways to say “no”* - *Block 4: Group work: Advertising posters*   *The following answer options are presented for each block:*  □ unsatisfactory □ rather unsatisfactory □ partly, partly □ rather satisfactory □ very satisfactory |  |
|  | Grading | 2 | **Did you use the students’ participation in the workshops or the poster creation for performance review/grading?**  ***□*** *yes*  *□ no* |  |
|  |  |  | *(Displayed only if “yes” chosen in the previous item)*  **Please briefly report for what you have been reviewed.** |  |

**Main questionnaire for Interventionists**

General note:

If not otherwise stated, response options range from

□ strongly disagree □ somewhat disagree □ partially agree, partially disagree □ somewhat agree □ fully agree

**Supplementary Table S6:***Interventionist questionnaires: measured constructs, number of items and example items translated in English*

| Part | Variable | Number of Items | Item / Validated scale | Sources |
| --- | --- | --- | --- | --- |
| A: Organizational information | School code | 1 | **School code** __________ |  |
|  | Grade | 1 | **Grade** ___________ |  |
|  | Class | 1 | **Class letter** ___________ |  |
|  | Initials | 1 | **Trainer's initials** (4 characters consisting of the first two letters of the first and last name) ___________ |  |
|  | Retrospective | 1 | **Did you fill out this questionnaire directly after the intervention (or in the same week), or was it a long time ago (retrospective)?**  □ directly after the intervention/within one week □ retrospective, intervention over one week ago |  |
|  | Class size | 1 | **How many children attend the class?** Dropdown: 0-30 **□** I don’t know |  |
|  | Number of children present | 1 | **How many children were present in class today?**  Dropdown: 0-30 **□** I don’t know |  |
|  | Unit | 1 | **Which unit was taught today?**  □ Core workshop 1 (90 min) □ Core workshop 2 (90 min)) □ Core workshop 1 and 2 (180 min) |  |
|  | Challenge | 1 | **If it was the second part of the core workshop: Which challenge was carried out?**  *(only displayed if core workshop 2 was chosen)*  □ Craft challenge □ Photo challenge |  |
|  | Norm message | 1 | **What norm message was given?**  *(only displayed if core workshop 2 was chosen)*  □ The majority of class x is smoke-free! (vs. tried smoking/vaping) □ The majority of class x is smoke-free! (vs. smokes regularly) □ The whole class x is smoke-free!  □ No message (too little consent) |  |
| B: Adherence (T1) | Duration | 2 | **How long was the entire session 1?**  □ Not taught today □ 0-10 □ 10-20 □ 20-30 □ 30-40 □ 40-50 □ 50-60 □ 60-70 □ 70-80 □ 80-90 □ 90-100 □ 100-110 □ 110-120 □ 120-130 □ 130-140 □ 140-150 □ 150-160 □ 160-170 □ 170-180  **How long was the entire session 2?** *(only displayed if core workshop 2 was chosen)*  □ Not taught today □ 0-10 □ 10-20 □ 20-30 □ 30-40 □ 40-50 □ 50-60 □ 60-70 □ 70-80 □ 80-90 □ 90-100 □ 100-110 □ 110-120 □ 120-130 □ 130-140 □ 140-150 □ 150-160 □ 160-170 □ 170-180 |  |
|  | Dose block-wise | 1 | **What percentage of the individual content was conveyed?**   - Health consequences - immediate effects, lungs, heart, blood vessels, experiment, cancer - Influence on aesthetics (appearance, smell), fitness in everyday life and athletic performance - Influence on the environment and passive smoking - Nicotine products and health - Tobacco company tricks, advertising and social media marketing (critical thinking) - Theory of saying no incl. joint collection - Act out saying no yourself as a trainer - Say no worksheet - Role play: Saying no - Dependence, influence on mental well-being and finances - Finances - Feedback on the questionnaire/smoking in class (norms) - Creative challenge (norms)   *The following answer options are presented for each block:*  □ 0-20% □ 20-40% □ 40-60% □ 60-80% □ 80-100% □ Not planned today |  |
| C: Competence (T1) | Overall quality | 1 | **The workshop went well.** | [35] |
|  | Quality components | 1 | **How satisfied were you with the teaching of the individual contents?**   - Health consequences - immediate effects, lungs, heart, blood vessels, experiment, cancer - Influence on aesthetics (appearance, smell), fitness in everyday life and athletic performance - Influence on the environment and passive smoking - Vapes, shishas and co and health - Tobacco company tricks, advertising and social media marketing (critical thinking) - Theory of saying no incl. joint collection - Act out saying no yourself as a trainer - Say no worksheet - Role play saying no - Dependence, influence on mental well-being and finances - Finances - Feedback on the questionnaire/smoking in class (norms) - Creative challenge (standards)     *The following answer options for each block:*  □ Unsatisfactory □ Rather unsatisfactory □ Partly, partly □ Rather satisfactory □ Satisfactory □ Not planned today |  |
|  | Quality of intervention delivery | 6 | **I actively listened to the students.** | Adapted from [36] |
| D: General report | Positive and negative events | 2 | **What positive events occurred while teaching the unit?** ____________ |  |
|  | Comments | 1 | **Any other comments?** ____________ |  |
| E: Adherence (T2) | Report booster - teacher | 1 | **After talking to the teacher and your own observation: What has the teacher already done in the booster/refresher unit?**   - Powerpoint presentation - Exchange of experiences saying no - Repetition/theory on strategies for saying no - Teacher has given standard message - Craft challenge with photos - Norm message visible on poster - Questionnaire completed by teacher     *The following answer options for each block:*  **□** yes □ no □ unknown |  |
|  | Report booster – study team | 1 | **Which of these components did you carry out?**  **Select “no” if not carried out because not planned/not possible (e.g. because the poster was missing/was there)**   - Marvel at the posters - Mindmap: Advantages of being smoke-free - Norm message picked up on poster - Norm message/feedback given on the survey itself - Future Plans - Sharing experiences Saying no - Worksheet: How to say no     *The following answer options for each block:*  **□** yes □ no □ |  |
|  | Duration booster – study team | 1 | **How long was the entire session? (Instruction, not including interview)**  □ 0-10 □ 10-20 □ 20-30 □ 30-40 □ 40-50 □ 50-60 □ 60-70 □ 70-80 □ 80-90 □ 90-100 |  |
|  | Dose block-wise: Booster – study team | 1 | **What percentage of the individual content was conveyed?**  *The first four options are only displayed if the interventionists have chosen them among the answers in the “Report booster- study team” block. We chose this setting because it was likely that those components would not be carried out in every intervention session, i.e., because the teacher may not have carried out the booster session. Thus, there may not always be posters to marvel at. In this case, our study team uses the mind-map as a substitute for the teacher-led repetition of workshop content. Furthermore, the norm feedback will only be given if there is a substantial amount of questionnaire responses for the class and there is an actual majority of smoke-free children.*   - Marvel at the posters - Take up norm feedback (see poster and take it up in the conversation with students) - Mind-map - Provide norm feedback   *Displayed every time:*   - Future Plans - Sharing experiences Saying no - Worksheet: How to say no     *The following answer options for each block:*  □ 0-20% □ 20-40% □ 40-60% □ 60-80% □ 80-100% |  |
| F: Competence (T2) | Overall quality | 1 | **The workshop went well.** | [35] |
|  | Quality components: Booster – study team | 1 | **How satisfied were you with the teaching of the individual contents?**   - Marvel at the posters - Take up standard feedback (see poster and take it up) - Mindmap - Provide standard feedback yourself   *The first four options are only displayed if the interventionists have chosen them among the answers in the “Report booster- study team” block (see above, “Dose block-wise”, for an elaboration on this setting)*  *Displayed every time:*   - Future Plans - Sharing experiences Saying no - Worksheet: How to say no     *The following answer options for each block:*  □ Unsatisfactory □ Rather unsatisfactory □ Partly, partly □ Rather satisfactory □ Satisfactory □ Not planned today/no poster available |  |
|  | Quality of intervention delivery | 6 | **I actively listened to the students.** | Adapted from [36] |
| G: Adherence (T3) | Duration | 1 | **How long was the entire session? (Instruction, not including interview)**  □ 0-10 □ 10-20 □ 20-30 □ 30-40 □ 40-50 □ 50-60 □ 60-70 □ 70-80 □ 80-90 □ 90-100 |  |
|  | Dose block-wise | 1 | **What percentage of the individual content was conveyed?**   - Block 1: Which things are good for me? - Block 2: Treasure chest - Block 3: Which situations can be difficult? - Block 4: If-Then-Worksheet - Block 5: Motivation Breathing (Ronaldo & Dandelion) - Block 7: Power card     The following answer options for each block:  **□ 0-20% □ 20-40% □ 40-60% □ 60-80% □ 80-100%** |  |
|  | Duration breathing exercise | 1 | **How many minutes of the breathing exercise were completed?**  □ 30 sec □ 1 min □ 1.5 min □ 2 min □ 2.5 min □ 2.5 min □ 3 min (100%) |  |
| H: Competence (T3) | Overall quality | 1 | **The workshop went well.** | [35] |
|  | Quality components | 1 | **How satisfied were you with the teaching of the individual contents?**   - Block 1: Which activities are good for me? - Block 2: Treasure chest - Block 3: Which situations can be difficult? - Block 4: If-Then-Worksheet - Block 5: Motivation Breathing (Ronaldo & Dandelion) - Block 6: Breathing exercise fish - Block 7: Power card     *The following answer options for each block:*  □ Unsatisfactory □ Rather unsatisfactory □ Partly, partly □ Rather satisfactory □ Satisfactory |  |
|  | Quality of intervention delivery | 6 | **I actively listened to the students.** | Adapted from [36] |

**References**

1. Stadler G, Chesaniuk M, Haering S, Roseman J, Straßburger VM, Martina S, et al. Diversified innovations in the health sciences: Proposal for a Diversity Minimal Item Set (DiMIS). Sustainable Chemistry and Pharmacy. 2023;33:101072.

2. Torsheim T, Cavallo F, Levin KA, Schnohr C, Mazur J, Niclasen B, et al. Psychometric Validation of the Revised Family Affluence Scale: a Latent Variable Approach. Child Indic Res. 2016;9:771–84.

3. Currie CE, Elton RA, Todd J, Platt S. Indicators of socioeconomic status for adolescents: the WHO Health Behaviour in School-aged Children Survey. Health Educ Res. 1997;12:385–97.

4. Goodman E, Adler NE, Kawachi I, Frazier AL, Huang B, Colditz GA. Adolescents’ perceptions of social status: development and evaluation of a new indicator. Pediatrics. 2001;108:E31.

5. Lampert T, Hoebel J, Kuntz B, Müters S, Kroll LE. Messung des sozioökonomischen Status und des subjektiven sozialen Status in KiGGS Welle 2. 2018;3.

6. Mang J, Seidl L, Schiepe-Tiska A, Tupac-Yupanqui A, Ziernwald L, Bias A, et al. PISA 2018 Skalenhandbuch. Dokumentation der Erhebungsinstrumente (2. Aufl.). 2023.

7. SOEP-Core - 2020: Jugend (11-17 Jahre, M3-M5, mit Verweis auf Variablen), SOEP Survey Papers 1128: Series A - Survey Instruments (Erhebungsinstrumente). Berlin: DIW Berlin/SOEP; 2022.

8. Robert Koch-Institut. Die allgemeine Gesundheit von Kindern und Jugendlichen in Deutschland – Querschnittergebnisse aus KiGGS Welle 2 und Trends. 2018. https://doi.org/10.17886/RKI-GBE-2018-004.

9. European Drug Abuse Prevention trial (EU-Dap) G. EU-Dap 1: Developing and Testing a New Drug Use Prevention Curriculum – 2003-2005. 2005. https://eudap.eu/eu-dap-1-developing-and-testing-a-new-drug-use-prevention-curriculum-2003-2005/. Accessed 3 Mar 2025.

10. Grard A, Schreuders M, Alves J, Kinnunen JM, Richter M, Federico B, et al. Smoking beliefs across genders, a comparative analysis of seven European countries. BMC Public Health. 2019;19:1321.

11. Chang H-Y, Wu W-C, Wu C-C, Cheng JY, Hurng B-S, Yen L-L. The incidence of experimental smoking in school children: an 8-year follow-up of the child and adolescent behaviors in long-term evolution (CABLE) study. BMC Public Health. 2011;11:844.

12. Brailovskaia J, Margraf J. How to measure self-esteem with one item? validation of the German single-item self-esteem scale (G-SISE). Curr Psychol. 2020;39:2192–202.

13. Dreiskaemper D, Tietjens M, Hohnemann S, Naul R, Freund P. PSK-Kinder – Ein Fragebogen zur Erfassung des physischen Selbstkonzepts von Kindern im Grundschulalter. Zeitschrift für Sportpsychologie. 2015;22:in press.

14. Kroenke K, Spitzer RL, Williams JBW, Löwe B. An ultra-brief screening scale for anxiety and depression: the PHQ-4. Psychosomatics. 2009;50:613–21.

15. Meade A, Craig B. Identifying Careless Responses in Survey Data. Psychological methods. 2012;17:437–55.

16. Ajzen MF Icek. Predicting and changing behavior: The reasoned action approach. New York: Psychology Press; 2009.

17. Montaño DE, Kasprzyk D. Theory of reasoned action, theory of planned behavior, and the integrated behavioral model. In: Health behavior: Theory, research, and practice, 5th ed. Hoboken, NJ, US: Jossey-Bass/Wiley; 2015. p. 95–124.

18. Choi WS, Gilpin EA, Farkas AJ, Pierce JP. Determining the probability of future smoking among adolescents. Addiction. 2001;96:313–23.

19. Pierce JP, Choi WS, Gilpin EA, Farkas AJ, Merritt RK. Validation of susceptibility as a predictor of which adolescents take up smoking in the United States. Health Psychology. 1996;15:355–61.

20. Strong DR, Hartman SJ, Nodora J, Messer K, James L, White M, et al. Predictive Validity of the Expanded Susceptibility to Smoke Index. Nicotine & Tobacco Research. 2015;17:862–9.

21. Kuntz B, Lampert T, KiGGS Study Group. Wasserpfeifenkonsum (Shisha-Rauchen) bei Jugendlichen in Deutschland. Bundesgesundheitsbl. 2015;58:467–73.

22. Lampert T. Smoking and Passive Smoking Exposure in Young People – Results of the German Health Interview and Examination Survey for Children and Adolescents (KiGGS). Deutsches Ärzteblatt international. 2008. https://doi.org/10.3238/arztebl.2008.0265.

23. Bäßler J, Mittag W. Selbstwirksamkeitserwartung im Umgang mit Drogen. In: Schwarzer R, Jerusalem M, editors. Skalen zur Erfassung von Lehrer- und Schülermerkmalen: Dokumentation der psychometrischen Verfahren im Rahmen der wissenschaftlichen Begleitung des Modellversuchs Selbstwirksame Schulen. Berlin: R. Schwarzer; 1999.

24. Heß F. Musikpräferenzen - Schüler [Fragebogenskala: Version 1.0]. In: Musikunterricht aus Schülersicht - Fragebogenerhebung (MASS 2011) [Skalenkollektion: Version 1.0]. Musikunterricht aus Schülersicht. 2017. https://doi.org/10.7477/350:197:1.

25. Kunter M, Schümer G, Artelt C, Baumert J, Klieme E, Neubrand M, et al. Selbstkonzept (Mathematik) - Schüler [Fragebogenskala: Version 1.0]. Programme for International Student Assessment (2000). 2012;Fragebogenerhebung Haupttest (PISA 2000) [Skalenkollektion: Version 1.0].

26. Sniehotta FF, Schwarzer R, Scholz U, Schüz B. Action planning and coping planning for long-term lifestyle change: theory and assessment. Eur J Soc Psychol. 2005;35:565–76.

27. Hrubá D, Žaloudíková I. Why to Smoke? Why not to Smoke? Major Reasons for Children’s Decisions on Whether or not to Smoke. Cent Eur J Public Health. 2010;18:202–8.

28. Vidrine JI, Anderson CB, Pollak KI, Wetter DW. Gender Differences in Adolescent Smoking: Mediator and Moderator Effects of Self-Generated Expected Smoking Outcomes. Am J Health Promot. 2006;20:383–7.

29. Murnaghan DA, Blanchard CM, Rodgers WM, LaRosa JN, MacQuarrie CR, MacLellan DL, et al. Predictors of physical activity, healthy eating and being smoke-free in teens: A theory of planned behaviour approach. Psychology & Health. 2010;25:925–41.

30. Schreiber M, Jenny GJ. Development and validation of the ‘Lebender emoticon PANAVA’ scale (LE-PANAVA) for digitally measuring positive and negative activation, and valence via emoticons. Personality and Individual Differences. 2020;160:109923.

31. Englert C, Bertrams A, Dickhäuser O. Entwicklung der Fünf-Item-Kurzskala STAI-SKD zur Messung von Zustandsangst. Zeitschrift für Gesundheitspsychologie. 2011;19:173–80.

32. Weichold K, Tomasik MJ, Silbereisen RK, Spaeth M. The Effectiveness of the Life Skills Program IPSY for the Prevention of Adolescent Tobacco Use: The Mediating Role of Yielding to Peer Pressure. Journal of Early Adolescence. 2016;36:881–908.

33. Lampert T, Kuntz B. Tabak- und Alkoholkonsum bei 11- bis 17-jährigen Jugendlichen. 2014. https://doi.org/10.25646/1821.

34. Grogan S, Conner M, Fry G, Gough B, Higgins A. Gender differences in smoking: A longitudinal study of beliefs predicting smoking in 11-15 year olds. Psychol Health. 2009;24:301–16.

35. Conner M, Grogan S, West R, Simms-Ellis R, Scholtens K, Sykes-Muskett B, et al. Effectiveness and cost-effectiveness of repeated implementation intention formation on adolescent smoking initiation: A cluster randomized controlled trial. Journal of Consulting and Clinical Psychology. 2019;87:422–32.

36. Cross W, West J, Wyman PA, Schmeelk-Cone K, Xia Y, Tu X, et al. Observational Measures of Implementer Fidelity for a School-based Preventive Intervention: Development, Reliability and Validity. Prev Sci. 2015;16:122–32.

**Supplementary Table S6:**

*Date and version identifier: revision chronology*

| Version |  | Comments, Amendments |
| --- | --- | --- |
| 2025-Mar-07 | Original |  |
| 2025-Oct-22 | Adaptation to formal checks | Additional detail clinical trial registration; provided CONSORT 2025 checklist |
| 2025-Mar-09 | Revision | Additional detail on development study procedure and results; Additional detail on trial measurements; minor structural changes; new Supplement (File 1: Development Study) |
